# Facile Manufacturing of PEEK‐Based Nanocomposites for High‐Efficiency Wide‐Temperature‐Range Electromagnetic Wave Absorption

**DOI:** 10.1002/advs.202523051

**Published:** 2025-11-26

**Authors:** Liang Zhao, Jingpeng Lin, Zifan Zheng, Yajun Chen, Xiong Yang, Yu Han, Yuchuan Cheng, Chenxi Hu, Zhihui Zeng

**Affiliations:** ^1^ Sino‐European Institute of Aviation Engineering Civil Aviation University of China Tianjin 300300 China; ^2^ State Key Laboratory of Coatings for Advanced Equipment Key Laboratory for Liquid–Solid Structural Evolution and Processing of Materials (Ministry of Education) School of Materials Science and Engineering Shandong University Jinan 250061 China; ^3^ College of Science Civil Aviation University of China Tianjin 300300 China; ^4^ Tianjin Istar‐Space Technology Co. Ltd. Tianjin 300300 China; ^5^ Laboratory of Atomic‐Scale and Micro & Nano Manufacturing Ningbo Institute of Materials Technology & Engineering Chinese Academy of Sciences Ningbo 315201 China

**Keywords:** electromagnetic wave absorption, high‐temperature performance, multifunctional composites, poly(ether ether ketone)

## Abstract

Developing high‐efficiency electromagnetic wave absorption with broad temperature adaptability in polymeric nanocomposites remains significant challenge despite urgent practical demands. Here, a new type of poly(ether ether ketone) (PEEK) composites integrated with magnetic carbon nanofillers of Fe_3_O_4_/carbon nanotubes/reduced graphene oxide are innovatively fabricated via the facile strategy. These multifunctional nanofillers collaboratively induce synergistic magnetic, conductive, and interfacial polarization losses, while maintaining structural integrity at high temperatures. Benefiting from this rational design, the resulting PEEK‐based composites exhibit outstanding absorption performance across 298–573 K. A minimum reflection loss (RL_min_) of −66.62 dB and an effective absorption bandwidth (EAB) of 4.58 GHz are achieved at room temperature, while a comparable performance involving RL_min_ of −66.01 dB and EAB_max_ of 4.05 GHz (8.20–12.25 GHz) is maintained even at 573 K, nearly covering the full X‐band, surpassing previously reported polymer composites at such a high temperature. This work opens up a novel pathway to engineer multifunctional polymer composites combining structural and electromagnetic functionalities at a wide temperature range.

## Graphical abstract

Herein, thermally robust poly(ether ether ketone)‐based composites reinforced by a hierarchical Fe_3_O_4_/carbon nanotubes/reduced graphene oxide (Fe_3_O_4_/CNTs/rGO) network are reported, delivering wide‐temperature‐range broadband electromagnetic wave absorption (298–573 K; minimum reflection loss (RL_min_) ≈ −66 dB). CNT‐involved heterogeneous interfaces dominate dielectric loss, while Fe_3_O_4_ provides ferromagnetic loss. Moreover, the additive‐manufacturing‐compatible platform paves the way for structural–functional integration in aerospace.

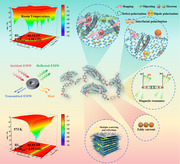

## 1 Introduction

With the rapid development of electronic devices and wireless communication technologies, electromagnetic wave (EMW) pollution has become one of the critical concerns, posing increasing threats to the proper functioning of precision instruments, human health, and data security.^[^ ^1^, ^2^, ^3^, ^4^ ^]^ To address this challenge, different potential materials have been explored as high‐performance EMW absorbing materials.^[^ ^5^, ^6^, ^7^ ^]^ Among various candidates, polymer‐based composites have attracted extensive attention due to their low density, excellent toughness, and relatively facile fabrication, offering great potential for achieving integrated structural and functional applications.^[^ ^8^, ^9^, ^10^ ^]^ By incorporating the commercial polymer matrices like epoxy resin,^[^ ^11^ ^]^ polydimethylsiloxane,^[^ ^12^ ^]^ polyvinylidene fluoride,^[^ ^13^ ^]^ and polyurethane,^[^ ^14^ ^]^ reinforced by various functional fillers, such as carbon materials, magnetic particles, and conductive polymers, have been widely studied and exhibited improved EMW absorption performance at ambient conditions. However, with the increasing demand of application environment, especially the wider temperature range, materials that only perform well under ambient conditions are no longer sufficient. When the operating temperature extends to a broad range (e.g., room temperature to 573 K), drawbacks have emerged in conventional polymers composites. The polymers tend to become soft, degrade, or lose mechanical integrity under high temperatures, disrupting the designed absorbing structures, breaking impedance matching, and ultimately leading to drastic performance deterioration or failure.^[^ ^15^ ^]^ Therefore, developing EMW absorbing polymeric composites with stable and efficient performance across wide temperature ranges remains an unmet challenge.

To meet the growing demand for wide‐temperature‐range EMW absorption, ceramic‐based composites have long been regarded as ideal candidates due to their exceptional thermal stability and mechanical integrity.^[^ ^16^, ^17^ ^]^ However, their complex processing requirements and high fabrication costs significantly hinder their competitiveness in mid‐range temperature applications, i.e., room temperature to 573 K. These limitations have prompted researchers to return to high‐performance polymers, such as poly(ether ether ketone) (PEEK) and polyimide, which exhibit favorable characteristics including low density, ease of processing, mechanical robustness, and excellent thermal resistance.^[^ ^18^, ^19^, ^20^, ^21^, ^22^ ^]^ These advantages make them physically suitable for medium to high‐temperature use. However, existing researches on these polymers primarily focused on their thermal and mechanical properties.^[^ ^23^ ^]^ Although several studies have been carried out to study their EMW absorption behaviors at ambient conditions,^[^ ^24^, ^25^, ^26^, ^27^ ^]^ rare attempt was devoted to reveal their high‐temperature EMW performance. The combination of adjustable‐functional fillers and polymer matrices with flexible processing technics for high‐performance EMW absorbers across wide temperatures is thus highly desired.^[^ ^23^, ^28^, ^29^, ^30^ ^]^

In this study, an innovative strategy that integrates a high‐performance PEEK matrix and synergistic multicomponent fillers to develop broadband, temperature‐tolerant EMW absorbing composites has been proposed. A ternary nanocomposite filler system comprising Fe_3_O_4_ nanoparticles, carbon nanotubes (CNTs), and reduced graphene oxide (rGO) was incorporated in PEEK that exhibits outstanding thermal and mechanical properties, achieving a synergy of multiple EMW loss mechanisms.^[^ ^31^, ^32^, ^33^, ^34^ ^]^ Specifically, Fe_3_O_4_ nanoparticles contribute ferromagnetic loss pathways, CNTs provide conductive networks to enhance conduction and interfacial polarization, and rGO introduces abundant interfaces and dipoles to promote dielectric loss and improve filler dispersion.^[^ ^35^ ^]^ Systematic investigations demonstrated that by optimizing filler type and content, the resulting composites exhibited exceptional and stable EMW absorption across 298–573 K. At room temperature, a minimum reflection loss (RL_min_) of −66.62 dB and a maximum effective absorption bandwidth (EAB_max_) of 4.58 GHz were achieved. Remarkably, even at 573 K, RL_min_ remained as low as −66.01 dB, and EAB_max_ reached 4.05 GHz, nearly covering the entire X‐band (8.2–12.4 GHz). These results significantly outperform previously reported polymer‐based EMW absorption systems. The superior broadband temperature response was attributed to the synergistic magnetic–carbon filler network and the structural integrity of PEEK composites, ensuring strong loss and good impedance matching across wide‐range temperatures. Additionally, the composite exhibited enhanced thermal and mechanical properties compared to pure PEEK. The designed composite also remains PEEK‐based composites’ structural robustness and stability that can be used for additive manufacturing. Therefore, this work provides a promising strategy for the development of PEEK‐based composites, capable of meeting the requirements of next‐generation multifunctional EMW absorbers operating across wide temperature ranges.

## 2 Results and Discussion

### 2.1 Structure and Morphology Characterization

**Figure 1:**
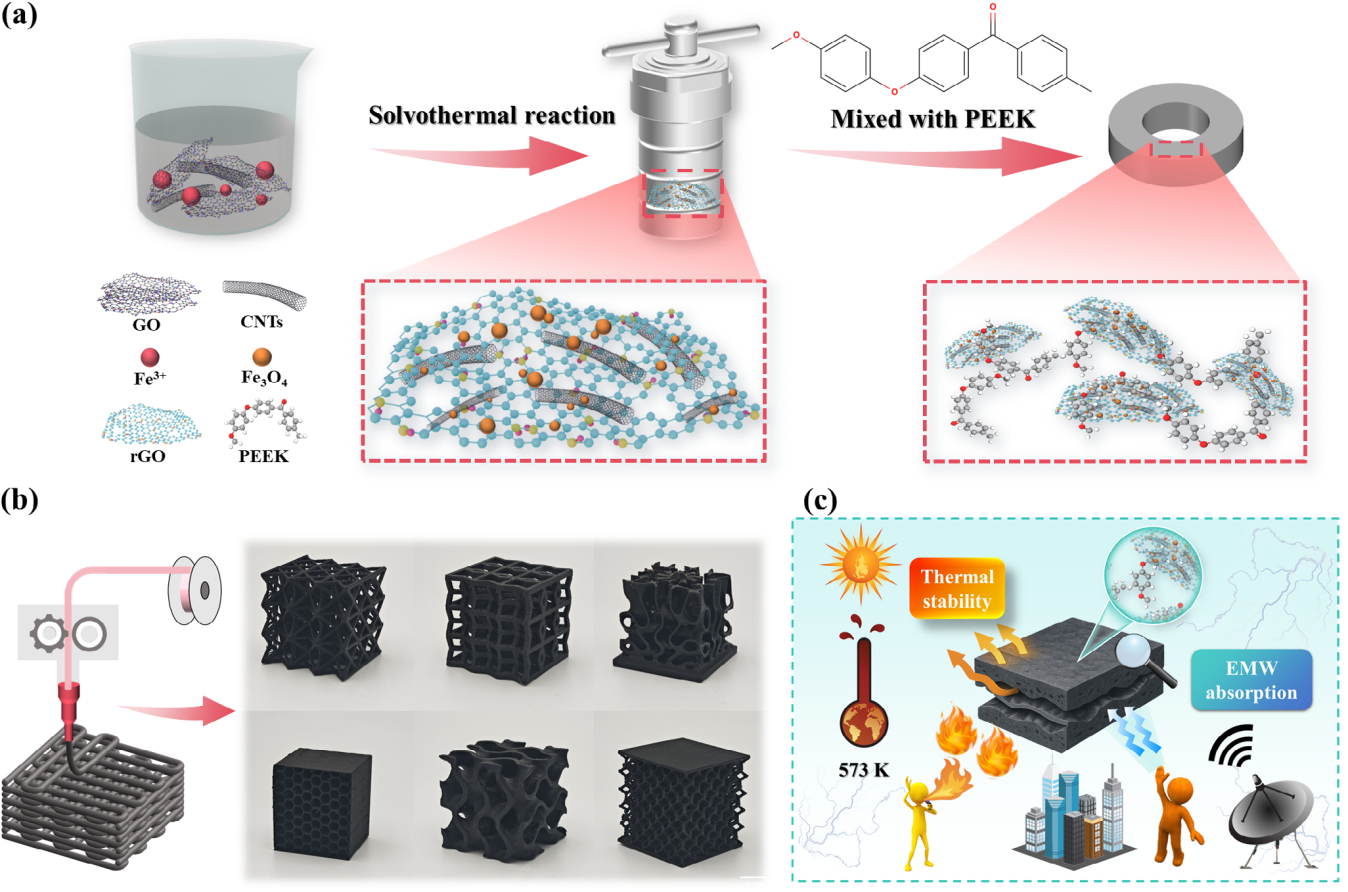
Facile fabrication illustration of Fe_3_O_4_/CNTs/rGO/PEEK (FCGP) composites. a) Schematic illustration of the fabrication process of FCGP composites. b) Structural samples based on 3D printing, demonstrating the additive manufacturing capability. c) Schematic diagram of FCGP composites for multifunctional applications.

The Fe_3_O_4_/CNTs/rGO/PEEK (FCGP) composites with efficient EMW absorption were fabricated by dispersing CNTs, GO, and Fe(NO_3_)_3_·9H_2_O in TEG, followed by a solvothermal method to form Fe_3_O_4_/CNTs/rGO (FCG) nanomaterials, which were subsequently blended with PEEK (**Figure** **1**a). After obtaining the FCGP composites, various physical products of different shapes can be fabricated through multiple processes such as hot pressing and 3D printing (Figure 1b). High‐performance FCGP composites can demonstrate excellent EMW absorption performance and thermal stability over a wide temperature range (Figure 1c). Such versatility in processing not only facilitates the integration of FCGP composites into complex device architectures but also endows them with great potential for practical applications in aerospace, flexible electronics, and electromagnetic protection. Moreover, the wide temperature adaptability ensures reliable performance in harsh service environments, opening new opportunities for next‐generation lightweight stealth technologies.

**Figure 2:**
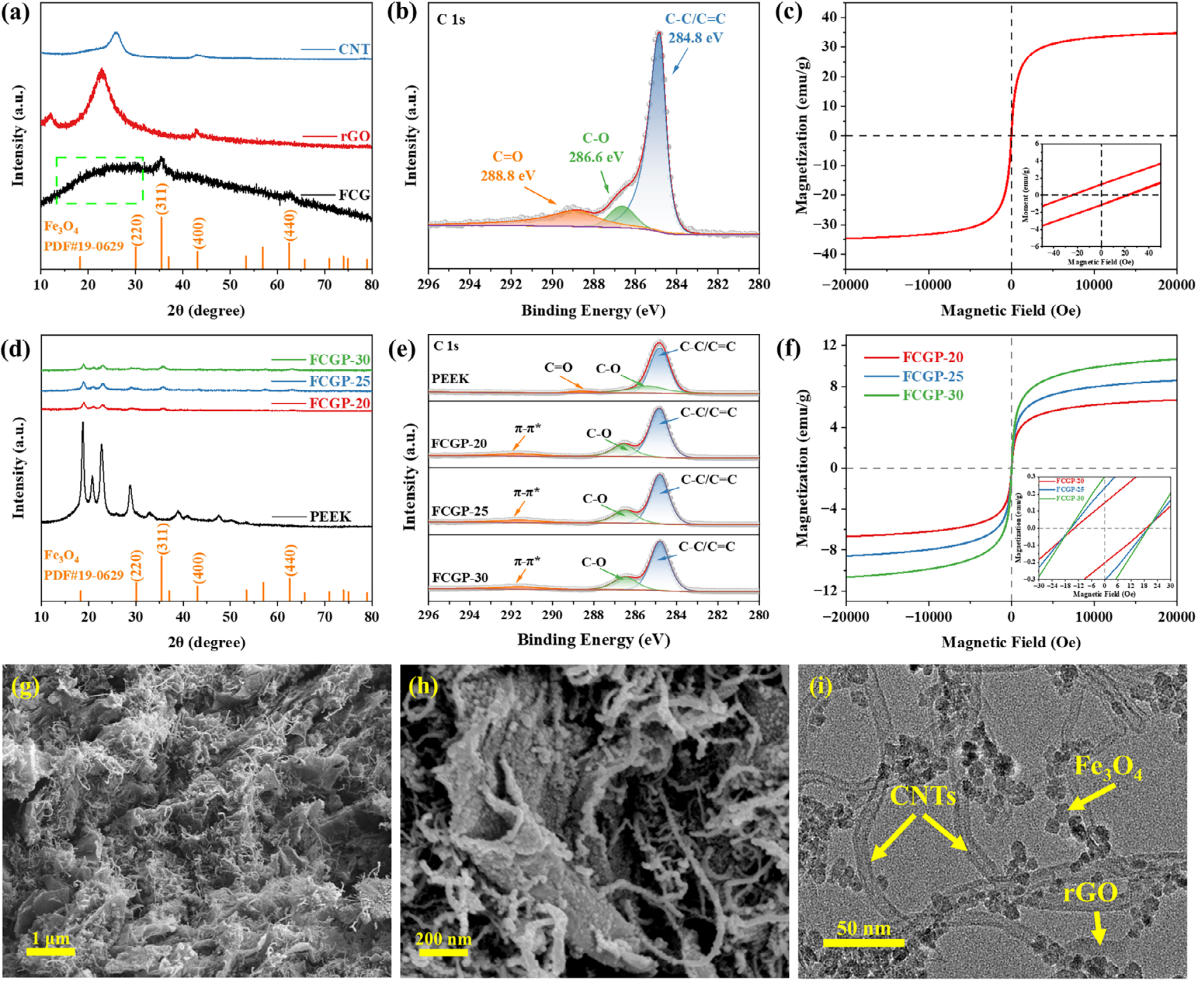
Microstructure characterizations and morphology. a) X‐ray diffraction (XRD) patterns of CNTs, rGO, and FCG nanomaterials b) X‐ray photoelectron spectroscopy (XPS) spectrum of C 1s region for FCG nanomaterials. c) Magnetic hysteresis loop of FCG nanomaterials. d) XRD patterns of PEEK and different FCGP composites. e) XPS spectrum of C 1s region for PEEK and FCGP composites. f) Magnetic hysteresis loop of FCGP composites. g,h) SEM images of FCG nanomaterials. i) TEM image of FCG nanomaterials.

X‐ray diffraction (XRD) is used to analyze the microstructure of materials. Compared with the characteristic peak of GO at 10.02° (Figure S1a, Supporting Information), corresponding to the (001) plane, the obvious peak disappears after solvothermal method, and is replaced by diffraction peaks at 22.93° and 42.88°, corresponding to the (002) and (100) planes, respectively. Such indicates that GO has been successfully reduced to rGO.^[^ ^36^ ^]^ The broadening of the peaks in the XRD pattern indicates the presence of disorder or defects in rGO, a result of incomplete crystallization in the reduction process.^[^ ^37^, ^38^ ^]^ The FCG nanomaterials show distinct diffraction peaks at 2*θ* of 30.09°, 35.42°, 43.05°, and 62.51°, corresponding to the (220), (311), (400), and (440) planes of Fe_3_O_4_ with a spinel structure (PDF#19‐0629), indicating that the Fe_3_O_4_ nanoparticles in the composite materials have good crystallinity (**Figure** **2**a).^[^ ^34^, ^39^ ^]^ XPS analysis confirmed the presence of C, O, and Fe in the FCG nanomaterials based on the survey scan spectrum (Figure S1b, Supporting Information). The C 1s XPS spectrum of the FCG nanomaterials shows peaks at 284.8, 286.6, and 288.8 eV, corresponding to C─C/C═C, C─O, and C═O, respectively (Figure 2b). The oxygen‐containing peaks indicate the presence of oxygen‐functional groups in rGO, which help prevent agglomeration between CNTs and rGO and provide abundant sites for Fe_3_O_4_ particle growth. Furthermore, polar bonds play a role in promoting dipole polarization under alternating electromagnetic fields.^[^ ^40^, ^41^ ^]^ The Fe 2p peak was deconvoluted into six peaks, with the two broad peaks at 711.4 and 724.9 eV corresponding to the Fe 2p_3/2_ and Fe 2p_1/2_ orbitals (Figure S1c, Supporting Information). Each orbital was further assigned to Fe^2+^ (710.8 and 724.2 eV) and Fe^3+^ (712.5 and 726.0 eV) spin–orbit states, and the binding energies of the satellite peaks for Fe^2+^ and Fe^3+^ are 720.4 and 731.9 eV, respectively, further confirming the presence of Fe_3_O_4_.^[^ ^42^ ^]^ The hysteresis loop of the FCG nanocomposite material was measured at room temperature using a superconducting quantum interference device magnetometer. The results show that the FCG nanomaterials exhibit typical ferromagnetism, with a saturation magnetization (*M*_s_) of 34.76 emu g^−1^, coercivity (*H*_c_) of 22.69 Oe, and remanent magnetization (*M*_r_) of 1.25 emu g^−1^ (Figure 2c). It is evident that the FCG nanocomposite material has low coercivity and saturation magnetization, with a small hysteresis loop area and a rapid response to the magnetic field, enabling it to efficiently absorb and transmit magnetic energy.^[^ ^43^ ^]^

As a semicrystalline polymer, the XRD pattern of PEEK typically displays four major diffraction peaks at 18.7°, 20.6°, 22.6°, and 28.6°, corresponding to the crystal planes (110), (111), (200), and (211) of PEEK, respectively.^[^ ^44^, ^45^, ^46^ ^]^ From the XRD spectra, it is evident that the intensity of the diffraction peaks at 35.42° and 62.51° gradually increases with the growth of FCG content, aligning well with the characteristic peaks of Fe_3_O_4_ in the FCG pattern (Figure 2d). In addition, full spectrum analysis reveals that pure PEEK exhibits characteristic peaks at 284.8 eV (C 1s) and 532 eV (O 1s), corresponding to the carbon backbone of the benzene ring and the oxygen atoms in the ether/ketone groups of its molecular chain. The FCGP composites show an additional Fe 2p peak around 710 eV, confirming the successful incorporation of Fe_3_O_4_ nanoparticles. C 1s spectra further reveal chemical bond reconstruction at the material interface: all samples exhibit a significant C─C/C═C bond peak at ≈284.8 eV (Figure 2e). Compared to pure PEEK, the binding energy of C─O bonds in the FCGP series samples shifts significantly from 285.4 to 286.5 eV, likely due to the formation of hydrogen bonds/coordination bonds between the ether bond oxygen in PEEK and the hydroxyl or oxygen‐containing functional groups on the surface of FCG nanomaterials, thus enhancing the heterointerface binding strength. Meanwhile, a distinct π–π* transition associated with sp^2^ hybridized orbitals is observed near 291.6 eV, which can be attributed to interfacial interactions between the FCG nanomaterials and the PEEK matrix. This suggests that when the hexagonal carbon rings of the FCG nanostructures face the phenyl rings of PEEK, π–π stacking interactions likely occur, forming chemical bonds and resulting in an enhanced peak intensity.^[^ ^47^, ^48^ ^]^ The O 1s spectra of FCGP composites and PEEK show that the FCGP samples exhibit Fe─O bonds and show an increase trend in C─O bond content and a decrease trend in C═O bond content compared to PEEK, which is consist with the C 1s spectra (Figure S1d, Supporting Information). All three FCGP samples exhibit significant ferromagnetism, with *M*_s_ values increasing from 6.68 to 10.71 emu g^−1^ as FCG content increases at room temperature (Figure 2f). It indicates that the magnetic effect of Fe_3_O_4_ magnetic particles outweighs the influence of nonmagnetic carbon. Moreover, the increase in *M*_s_ leads to a higher magnetic loss intensity, optimizing the magnetic properties of the material and contributing to EMW attenuation.^[^ ^49^ ^]^ The corresponding *H*_c_ values are 18.75, 19.71, and 19.84 Oe, showing slight fluctuations, which could be attributed to the material's microstructure and the size of the Fe_3_O_4_ particles.^[^ ^50^, ^51^ ^]^

At low magnification, 1D CNTs are uniformly dispersed on 2D rGO layers, exhibiting a cross‐linked structure without noticeable aggregation. The CNTs are tightly embedded within the layered rGO framework, establishing stable heterointerfaces. Fe_3_O_4_ nanoparticles are homogeneously distributed on the surfaces of CNTs and rGO, constructing a 3D network (Figure 2g). With increasing magnification, the tubular structure of the CNTs becomes more distinct, while defect sites on their surfaces serve as nucleation sites for Fe_3_O_4_ nanoparticles (Figure 2h). Both oxygen and iron elements exhibit a relatively uniform distribution within the composite material (Figure S2a–c, Supporting Information). TEM images are further employed to analyze the microstructure of the Fe_3_O_4_/CNTs/rGO composite. Fe_3_O_4_ nanoparticles are uniformly dispersed on the CNT surfaces. Owing to their small size, Fe_3_O_4_ nanoparticles exhibit a tendency to agglomerate (Figure 2i). At a higher magnification, the crystalline structure of Fe_3_O_4_ nanoparticles is distinctly observed, with an interplanar spacing of 0.292 nm corresponding to the (220) plane of Fe_3_O_4_, in agreement with the XRD results (Figure S2d, Supporting Information).

### 2.2 EMW Absorption Performance of FCGP Composites at Room Temperature

**Figure 3:**
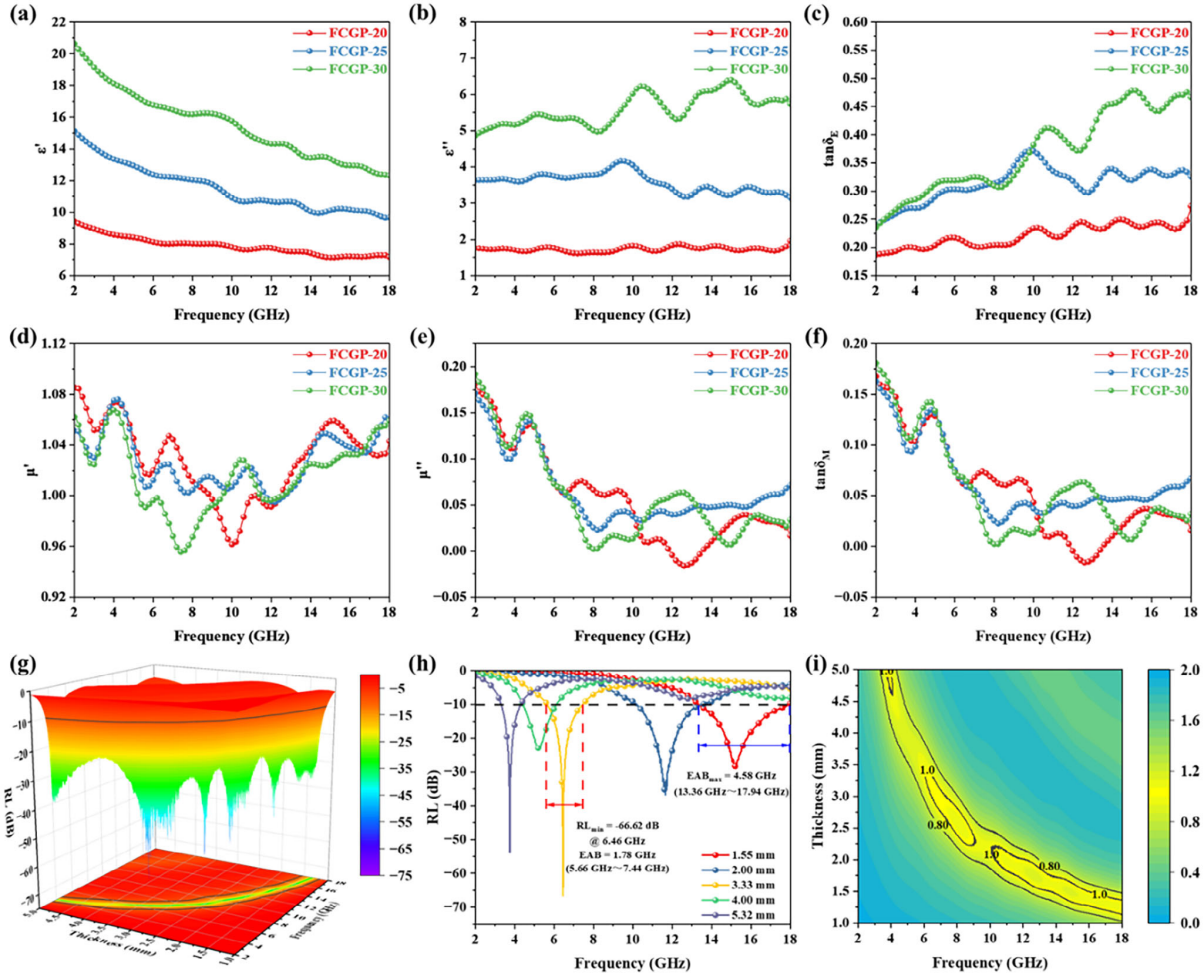
Electromagnetic parameters analysis and EMW absorption performance. a) The real part (*ε′*) and b) imaginary part (*ε″*) of permittivity for FCGP composites. c) Dielectric loss tangent (tan*δ*_E_) for FCGP composites. d) The real part (*µ′*) and e) imaginary part (*µ″*) of permeability for different FCGP composites. f) Magnetic loss tangent (tan*δ*_M_) for different FCGP composites. g) 3D RL curves of FCGP‐25 (1–5 mm). h) 2D RL curves of FCGP‐25. i) Impedance matching characteristics of FCGP‐25.

The real part (*ε′*) of permittivity shows an overall decreasing trend with frequency due to frequency dispersion effect (**Figure** **3**a).^[^ ^52^, ^53^, ^54^ ^]^ Within the 2–18 GHz frequency range, the complex permittivity of the composites increases with increasing FCG content. This enhancement arises from the formation of more continuous conductive networks and intensified interfacial polarization, which facilitate the formation of additional conductive pathways within the composite, thereby converting EMW energy into heat.^[^ ^55^, ^56^, ^57^, ^58^ ^]^ Additionally, heterogeneous interfaces are present both within the FCG structure and at the interfaces between FCG and PEEK matrix. These abundant heterointerfaces facilitate charge accumulation within the filler as well as at the filler–matrix boundary. With increasing FCG content, the number of these interfaces rises, enhancing the interfacial polarization effect and consequently elevating the permittivity.^[^ ^59^ ^]^ The peaks observed on the imaginary part of permittivity signify enhance EMW absorption at specific frequencies (Figure 3b). These peaks originate from various mechanisms, including charge accumulation and relaxation at nonuniform interfaces, dipolar polarization relaxation under external electric fields, and energy dissipation due to free charge migration. It is crucial to recognize that interpreting the peaks in the curves is complex, as a single loss mechanism can give rise to multiple peaks, while a single peak may result from the superposition of multiple loss mechanisms. For instance, polarization relaxation and conduction losses can simultaneously occur over a given frequency range, leading to a broad peak or multiple overlapping peaks within that range.

In the entire frequency range, the complex permeability exhibits several fluctuations (Figure 3d). Some resonance peaks can be found in the *µ″*–*f* curve, indicating the presence of multiple loss mechanisms in the composites, including natural resonance, exchange resonance, and eddy current loss. At the natural resonance frequency, the imaginary part of the complex permittivity or permeability exhibits a significant increase. For instance, the resonance peak near 5 GHz in the *µ″*–*f* curve suggests that this frequency corresponds to the material's inherent resonant frequency, which aligns with a sharp decline in the real part of permeability (Figure 3e). By comparison, it can be found that tan*δ*_E_ is greater than tan*δ*_M_ throughout the entire band, proving that in the attenuation of EMW, the dielectric loss of FCGP material is dominant and the magnetic loss is auxiliary (Figure 3c,f).

In all 3D RL plots presented in this work, the solid black line delineates the effective absorption region when RL is less than −10 dB. FCGP‐20 shows RL_min_ of only −16.65 dB (5.00 mm) with a maximum EAB of 2.62 GHz (1.73 mm) (Figure S3a1,a2, Supporting Information). Thus, FCGP‐20 does not exhibit satisfactory performance. However, with an increased filler ratio, the as‐prepared composite's performance improves significantly. When the frequency is 6.46 GHz, RL_min_ of FCGP‐25 reaches −66.62 dB (3.33 mm). The EAB_max_ reaches 4.58 GHz (13.36–17.94 GHz, 1.55 mm) (Figure 3g). From the projection map, it can be seen that FCGP‐25 covers the C, X, and Ku bands within the 2–18 GHz frequency range, and part of S‐band EMW can also be absorbed, indicating outstanding wave‐absorbing performance. It could also be found that the maximum EAB of the material is located in the high‐frequency region, with the effective absorption bandwidth generally narrowing as the frequency decreases. Notably, at the matching thickness (3.33 mm) that corresponds to the maximum absorption peak, EAB of FCGP‐25 reaches 1.78 GHz (5.66–7.44 GHz). Additionally, when the thickness is 5.32 mm and the frequency is 3.76 GHz, the RL is less than −50 dB, achieving 99.999% EMW absorption (Figure 3h). These results indicate that FCGP‐25 still exhibits good absorption performance and EAB in the low‐frequency range and even surpasses typical EMW absorbers ever reported.^[^ ^60^, ^61^ ^]^ By contrast, FCGP‐30 has a RL_min_ of −74.45 dB (2.33 mm, 8.18 GHz) and a maximum EAB of 4.38 GHz (13.58–17.96 GHz, 1.33 mm) (Figure S3b1,b2, Supporting Information). Considering the comprehensive characteristics of wave‐absorbing materials, FCGP‐25 is selected for further investigation and testing.

To further study the EMW absorption performance of PEEK‐based composites, we changed the filler type while keeping the same filler ratio as FCGP‐25. The results show that FCP‐25 (Fe_3_O_4_/CNTs/PEEK) has a RL_min_ value of −59.21 dB at 7.28 GHz and a maximum EAB of 3.84 GHz (13.92–17.76 GHz, 1.32 mm), demonstrating high EMW absorption performance (Figure S3c1,c2, Supporting Information). The projection map corresponding to the 3D plot also shows that the absorption range of this sample in the 2–18 GHz frequency range is similar to FCGP‐25, effectively absorbing the S (partial), C, X, and Ku bands. However, the overall absorption intensity and EAB are slightly inferior to those of FCGP‐25. When the EMW absorption agent was changed to FGP‐25, the performance was worse, with 90% or more EMW absorption only achieved in the 5.97–8.29 GHz range (Figure S3d1,d2, Supporting Information). When Fe_3_O_4_ nanoparticles were incorporated into the PEEK matrix, FP‐25 (Fe_3_O_4_/PEEK) has a RL_min_ of −24.87 dB and EAB_max_ of 6.25 GHz, effectively covering the Ku band (Figure S3e1,e2, Supporting Information). All the absorption performance statistics of the PEEK‐based wave‐absorbing composite prepared in this study are shown in Table S1 (Supporting Information). In summary, FCGP‐25 exhibits the best wave absorption performance among all the samples. Moreover, these results strongly demonstrate the compatibility between the PEEK matrix and various absorbing agents. Evidently, by adjusting the type and content of the absorbing agent, the electromagnetic parameters of the composite can be effectively tuned, ultimately controlling the EMW absorption performance.

To better explain the differences in performance, impedance matching was introduced to evaluate the EMW entering performance of the samples. FCGP‐25 demonstrates good impedance matching, with its *Z* values clearly between 0.8–1.2 range, which correlates well with the RL ≤ −10 dB region (Figure 3i). For FCGP‐20, FCGP‐30, FCP‐25, FGP‐25, and FP‐25, it is evident that regions where the *Z* value is close to 1 are also the areas with excellent EMW absorption performance (Figure S3a3,b3,c3,d3,e3, Supporting Information). These results emphasize that optimal impedance matching is essential for EMW to effectively penetrate the material.

**Figure 4:**
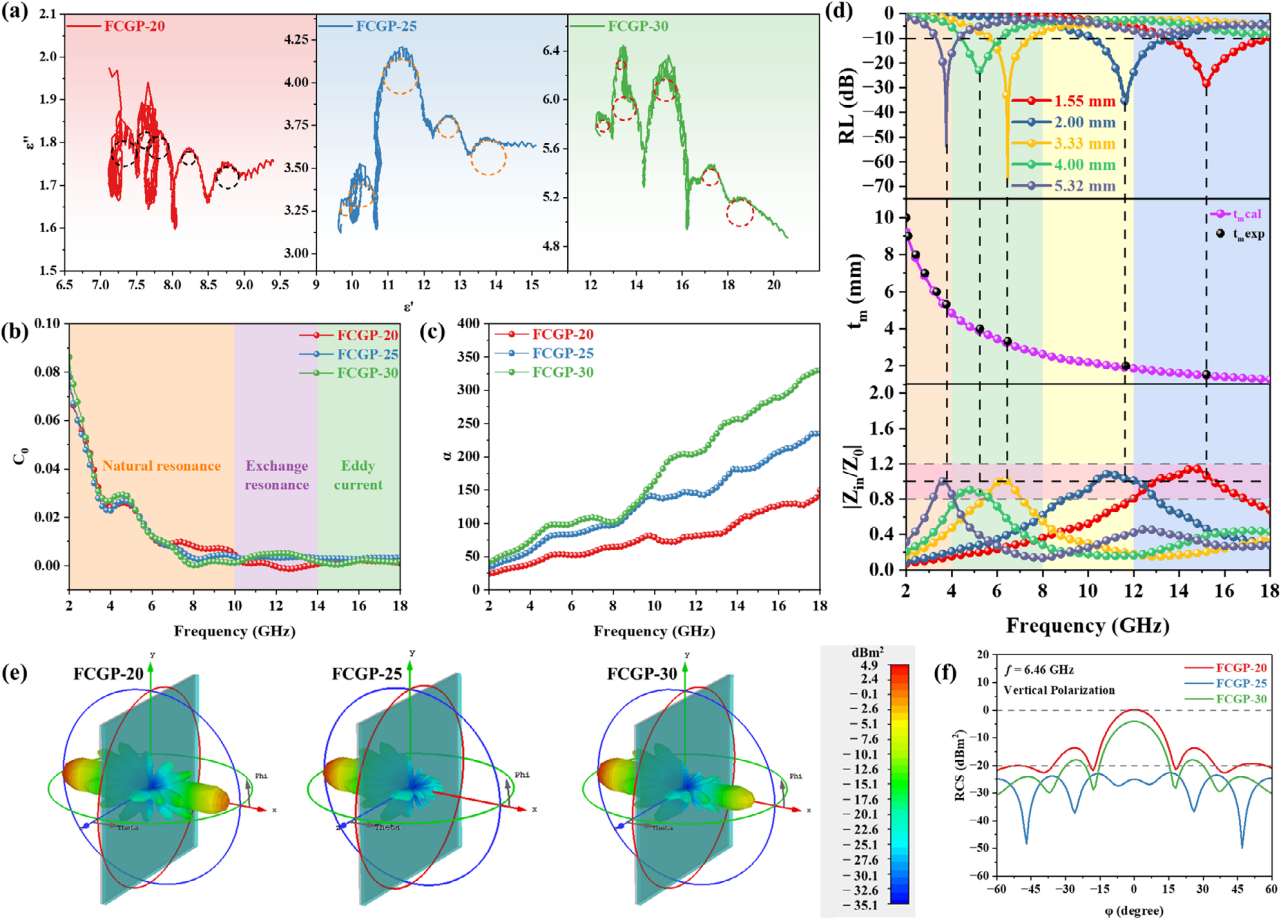
EMW absorption mechanisms and performance comparisons of FCGP composites. a) The Cole–Cole plots of different FCGP composites. b) The *C*_0_ curves of different FCGP composites. c) The attenuation coefficient of different FCGP composites. d) Reflection loss–frequency curves, relationship between matching thickness and theoretic quarter‐wavelength matching thickness, and impedance matching of FCGP‐25 composites. e) 3D radar cross‐section (RCS) simulation results of FCGP composites. f) 2D RCS simulation results of FCGP composites within the angular range of −60° < *φ* < 60°.

In the Cole–Cole plot, it can be observed that FCGP‐30 exhibits more semicircles than FCGP‐20 and FCGP‐25 (**Figure** **4**a). Moreover, the appearance of the Cole–Cole semicircles correlates with the frequencies at which the permittivity changes. This relationship can be easily observed by plotting the permittivity versus frequency in a 3D scatter plot (Figure S4, Supporting Information). For FCGP‐25, the three most prominent semicircles appear at ≈9.5, 13.7, and 15.8 GHz, which correspond precisely to the frequencies where the imaginary part of the permittivity peaks (Figure 3b). This suggests that the peaks are caused by polarization relaxation losses at specific frequencies. Notably, as the FCG content increases, the slope of the straight line at the curve's tail gradually decreases, indicating that the conductive loss in the high‐frequency region is relatively small. According to free electron theory, polarization loss is proportional to the conductivity.^[^ ^62^ ^]^ It can be observed that conductive loss decreases monotonically with increasing frequency, while polarization loss remains higher than conductive loss (Figure S5, Supporting Information). To identify the interfaces dominating the high dielectric loss, we compared the frequency‐dependent dielectric parameters of three systems with progressively richer interfacial architectures FP‐25, FCP‐25, and FGP‐25 (Figure S6, Supporting Information). FCP‐25 exhibits the largest *ε′* and the highest *ε″* accompanied by multiple relaxation features whereas FP‐25 and FGP‐25 display fewer and weaker features. The greater number and breadth of *ε″* relaxations in FCP‐25 indicate stronger polarization arising from hierarchical heterogeneous interfaces, in particular Fe_3_O_4_/CNTs and CNTs/PEEK, in addition to Fe_3_O_4_/PEEK. By contrast, FP‐25 contains only the Fe_3_O_4_/PEEK interface, and FGP‐25 mainly involves Fe_3_O_4_/rGO and rGO/PEEK interfaces, leading to weaker interfacial polarization. Moreover, we also analyzed the Cole–Cole plots of three systems with progressively richer interfacial architectures, which further corroborate these results (Figure S7, Supporting Information). FCP‐25 shows the most complex Cole–Cole pattern composed of numerous overlapping semicircles, evidencing multiple relaxation processes and a broad distribution of relaxation times. These results indicate strong polarization originating from hierarchical interfaces involving CNTs (Fe_3_O_4_/CNTs and CNTs/PEEK) in addition to Fe_3_O_4_/PEEK. By contrast, FP‐25 with a single interface and FGP‐25 with sheet‐like rGO interfaces exhibit fewer relaxations and weaker polarization. Therefore, the high dielectric loss is predominantly governed by CNT‐related interfaces through intensive interfacial charge accumulation and relaxation, while Fe_3_O_4_ provides ferromagnetic loss via natural/exchange resonance. Beyond furnishing additional interfaces, rGO plays a critical role in regulating filler dispersion. For FCGP‐25, the 2D rGO sheets act as conductive bridges and spacers, which mitigate CNT bundling and promote a uniformly percolated CNT network throughout the PEEK matrix. Clearly, the *C*_0_ values of the three samples are not straight lines, indicating that the magnetic loss mechanism of the composite materials is not solely due to eddy current loss (Figure 4b). The natural resonance peaks (≈2–10 GHz) and exchange resonance peaks (≈10–14 GHz) can be observed, and in the high‐frequency region (14–18 GHz), the *C*_0_ value remains almost constant, suggesting that eddy current loss dominates in this frequency range.^[^ ^63^ ^]^

As the FCG content increases, the attenuation coefficient (*α*) values of FCGP composites significantly increase, which can be ascribed to the mentioned multiple polarization relaxation, conductivity loss, and magnetic losses (Figure 4c). However, although the attenuation coefficient of FCGP‐30 is higher than FCGP‐25 and FCGP‐20, the worse wave absorption performance could be ascribed to the impedance mismatch. Additionally, the maximum attenuation coefficient for each sample occurs at high frequencies, while the RL_min_ for all samples does not occur in the high‐frequency region, which suggests that the EMW absorption performance not only results from the magnitude of the attenuation coefficient, but good impedance matching is also key to achieving effective EMW absorption. Therefore, FCGP‐25 achieves the best EMW absorption performance by balancing the impedance matching and attenuation coefficient. Furthermore, the matching thickness reduces with the optimal absorption peak moves toward higher frequencies (Figure 4d), suggesting that the quarter‐wavelength theory also contributes to the attenuation of electromagnetic waves. For FCGP‐25, when *f*_m_ = 6.46 GHz, the theoretical matching thickness *t*_m_ = 3.20 mm, while the experimentally measured optimal thickness is 3.33 mm, with a deviation of 4%, possibly due to material nonhomogeneity or interface scattering effects. At the optimal matching thickness, the *Z* value of FCGP‐25 is very close to 1.0, achieving more than 99.9999% wave absorption. A similar trend can be observed in all other samples (Figure S8, Supporting Information).

To further investigate the radar stealth performance of FCGP‐25 in practical applications, the radar cross‐section (RCS) of FCGP samples was simulated using CST Microwave Studio software (Figure S9, Supporting Information). At 6.46 GHz, when the thickness is 3.33 mm, FCGP exhibits various 3D RCS values (Figure 4e). FCGP‐25 has a RCS value of less than −20 dBm^2^ over most of the angular range. The results of the 2D RCS values further highlight the advantages of FCGP‐25, with its RCS value consistently remaining below −20 dBm^2^ over a wide angular range, demonstrating the best stealth effect compared to the other two samples (Figure 4f).

### 2.3 High‐Temperature EMW Absorption Performance

**Figure 5:**
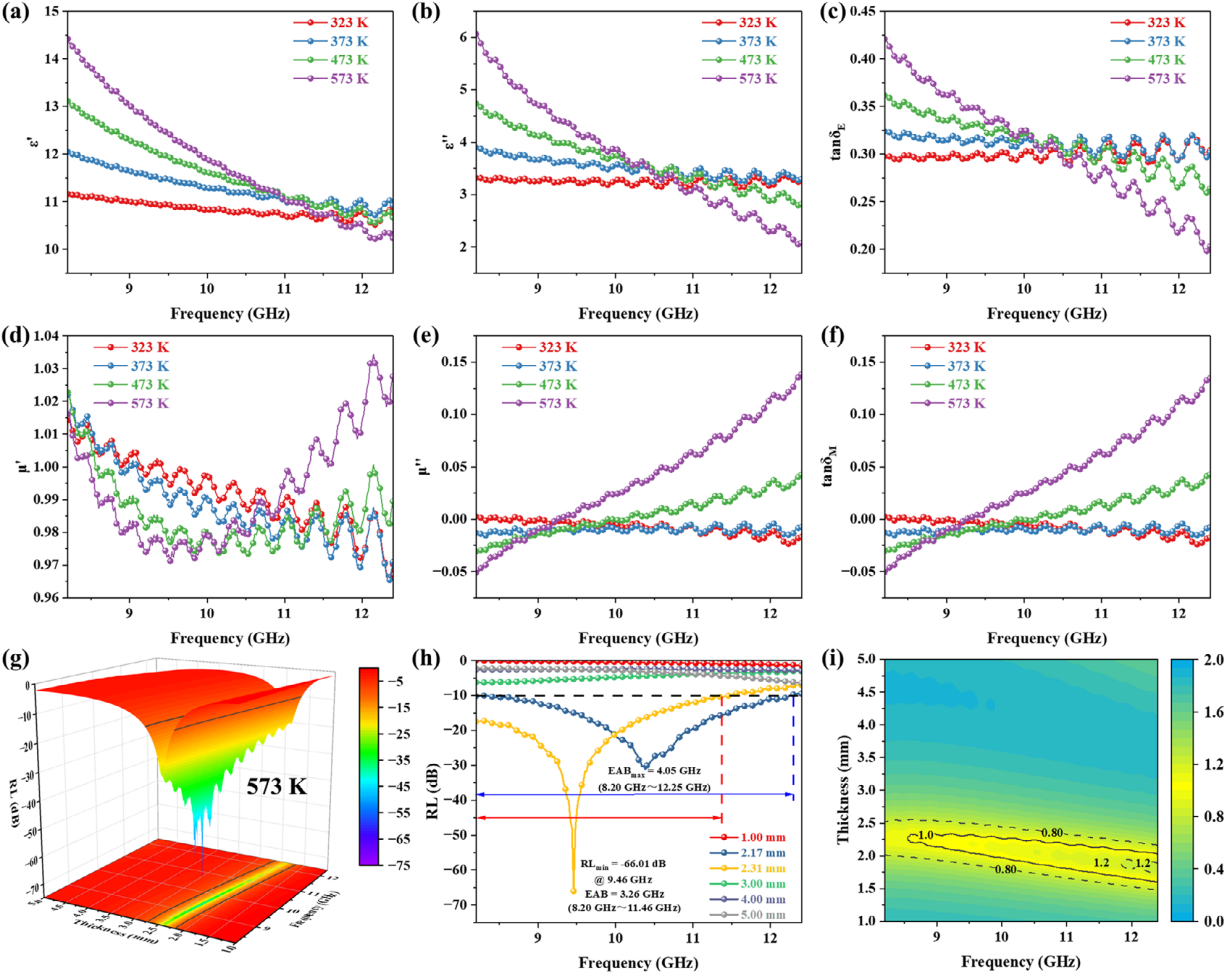
Electromagnetic parameters’ analysis and EMW absorption performance at different temperatures. a) The real part (*ε′*) and b) imaginary part (*ε″*) of permittivity for FCGP‐25. c) Dielectric loss tangent (tan*δ*_E_) for FCGP‐25. d) The real part (*µ′*) and e) imaginary part (*µ″*) of permeability for FCGP‐25. f) Magnetic loss tangent (tan*δ*_M_) for FCGP‐25. g) 3D RL curves of FCGP‐25 (1–5 mm) at 573 K. h) 2D RL curves of FCGP‐25 at 573 K. i) Impedance matching characteristics for FCGP‐25 at 573 K.

To evaluate the EMW absorption performance under wider temperature range, the complex permittivity and permeability of the composites in the X band range (8.2–12.4 GHz) were measured using a wave‐guide method. At 323 K, *ε′* and *ε″* of the permittivity of FCGP‐25 are ≈10.8 and 3.2, respectively (**Figure** **5**a,b). With increasing temperature from 323 to 573 K, both *ε′* and *ε″* exhibit a rising trend, indicating a temperature‐sensitive dielectric behavior, which can be interpreted using Debye relaxation theory. Meanwhile, the permittivity increases with temperature under the same frequency, which can be attributed to two main factors: first, higher temperatures accelerate molecular motion, promoting orientation polarization of polar groups under the influence of the alternating electric field, thereby enhancing dielectric loss; second, elevated temperatures facilitate electron transitions, leading to increased conductive loss.^[^ ^26^ ^]^ Additionally, it is evident that the positive correlation between temperature and the permittivity weakens with increasing frequency. This can be attributed to the following mechanism: at low frequencies (*ωτ* ≪ 1), the electric field changes slowly, allowing sufficient time for polarization to occur. As temperature rises, the relaxation time *τ* decreases significantly, leading to faster polarization and thereby increasing both *ε′* and *ε″*. For instance, when the temperature increases from 323 to 573 K, *ε′* and *ε″* of FCGP‐25 at 8.2 GHz rise by 29.2% and 82.8%, respectively. In the high‐frequency region (*ωτ* ≈ 1 or ≫ 1), the rate of field variation approaches or exceeds the intrinsic polarization frequency (1/*τ*), preventing polarization from keeping up with the rapidly oscillating electric field, thus reducing temperature sensitivity. The temperature‐dependent behavior of permittivity, as the temperature increases from 323 to 573 K, clearly illustrates this trend (Figure S10, Supporting Information). Therefore, the dielectric loss tangent increases with rising temperature before 10.5 GHz, while the temperature sensitivity of the dielectric constant weakens after that. This phenomenon results from the combined effect of thermal activation and the time‐varying nature of the electromagnetic field on dielectric response (Figure 5c). The real part of permeability of FCGP‐25 remains within the range of 0.96–1.04 at different temperatures, indicating weak magnetism (Figure 5d). Noticeable fluctuations are present, which are attributed to natural and exchange resonance effects. Additionally, the imaginary part *µ″* exhibits negative values under certain temperature and frequency conditions (Figure 5e). This phenomenon is attributed to the material′s high electrical conductivity, which promotes the formation of numerous microscopic conductive networks. These networks generate induced currents, producing secondary magnetic fields that interact with the externally applied magnetic field and radiate magnetic energy outward. At room temperature, a similar but weaker response observed for FCGP‐20 is mainly attributed to localized conductive paths and interfacial eddy currents.^[^ ^64^, ^65^, ^66^ ^]^ The negative *µ″* observed at high temperatures originates from enhanced electrical conductivity and thermally induced current loops, which radiate magnetic energy outward. Therefore, although *µ″* < 0 appears in both conditions, the high‐temperature mechanism involves additional carrier excitation and magnetization relaxation effects.^[^ ^67^, ^68^ ^]^ In the *C*_0_–*f* curves of the material under different temperatures, the absence of linearity suggests that magnetic loss originates from multiple ferromagnetic resonance mechanisms within the measured frequency range (Figure S11, Supporting Information). Comparative analysis indicates that elevated temperatures moderately enhance magnetic losses, which become more pronounced at higher frequencies (Figure 5f). However, the overall contribution of magnetic loss remains lower than that of dielectric loss.

Across this temperature range, the RL_min_ and the EAB_max_ of FCGP‐25 increase. Specifically, the RL_min_ is −19.62 dB and EAB_max_ is 2.58 GHz at 323 K (Figure S12a1,a2, Supporting Information). When the temperature rises to 373 K, the EMW absorption improves slightly to −22.99 dB (over 99% absorption), and EAB_max_ expands to 2.84 GHz (Figure S12b1,b2, Supporting Information). At 473 K, RL_min_ reaches −34.64 dB and EAB_max_ increases to 3.34 GHz (Figure S12c1,c2, Supporting Information). It is noteworthy that RL_min_ consistently appears at 8.22 GHz for all three temperatures, indicating a stable absorption frequency, while the increase of the attenuation constant *α* from ≈85 to ≈120 suggests enhanced attenuation ability with rising temperature (Figure S13, Supporting Information). Furthermore, the EAB_max_ values occur at comparable thicknesses and show a trend toward lower frequencies. At 573 K, RL_min_ reaches −66.01 dB (2.31 mm) (Figure 5g). The EAB also shifts further toward lower frequencies and broadens significantly to 4.05 GHz (8.20–12.25 GHz), nearly covering the entire test band (Figure 5h). When the temperature rises from 373 to 473 K, the impedance matching of FCGP‐25 improves significantly, as the *Z* values decrease from 1.39 and 1.34 to ≈1.2 (Figure S12a3,b3,c3, Supporting Information). At 573 K, the *Z* values remain within the range of 0.95–1.2 across the entire testing frequency range, indicating excellent impedance matching (Figure 5i). Thus, the outstanding performance observed at 573 K results from a well‐balanced interplay between impedance matching and EMW loss. The high‐temperature stability of absorption mainly results from the conductive and magnetic networks established by FCG fillers, while the PEEK matrix provides a thermally stable structural framework. Notably, PEEK itself is an almost nonabsorbing resin material. For neat PEEK, the real part (*µ′*) and imaginary part (*µ″*) of the complex permeability are independent on the frequency in the investigated range. The calculated 3D RL curves confirm that its EMW performance is negligible, indicating that the fillers play the dominant role in maintaining efficient absorption at elevated temperatures (Figure S14, Supporting Information).

Overall, the EMW absorption performance of FCGP‐25 improves significantly with increasing temperature, as evidenced by both the enhanced RL_min_ and the expanded EAB_max_ (Figure S15, Supporting Information). Moreover, the improvement in RL_min_ is accompanied by a reduction in the matching thickness, which is a favorable trend for high‐temperature EMW absorbers. These excellent thermal resistances and EMW absorption properties make FCGP‐25 a promising candidate for high‐temperature stealth applications.

### 2.4 EMW Absorption Mechanism

**Figure 6:**
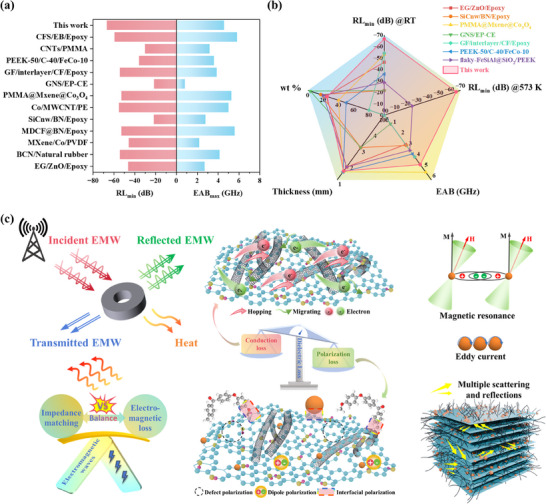
a) Comparison of RL_min_ and EAB_max_ performance between FCGP‐25 and other polymer‐based EMW absorbers. b) Comprehensive performance comparison of FCGP‐25 with other polymer‐based EMW absorbers. c) Schematic illustration of EMW absorption mechanisms for FCGP composites.

**Figure** **6**a shows a comparison of the FCGP‐25 with previously reported polymer‐based EMW absorbers, focusing on key indicators such as the RL_min_ and EAB_max_. Notably, the FCGP‐25 composite demonstrates impressive performance over previously reported polymer‐based EMW absorbers (Figure 6a,b), achieving a low RL and a broader EAB at a thin matching thickness and low filler content. This exceptional performance is attributed to the synergistic effects of dielectric and magnetic losses, as well as the optimized impedance matching and attenuation capability. Table S2 (Supporting Information) provides the detailed performance data of these absorbing materials.

In summary, Fe_3_O_4_ nanoparticles, rGO, and CNTs in the fillers form a crucial 3D conductive network, enabling free electrons to undergo hopping and migration under an alternating electric field. This effectively converts incident electromagnetic energy into heat, which is subsequently dissipated. Importantly, by adjusting the filler loading, the synergistic interaction between the PEEK matrix and FCG optimizes the material's impedance matching, thereby facilitating greater penetration of EM waves into the material. Additionally, abundant heterogeneous interfaces exist within the composite, where unevenly distributed positive and negative charges accumulate, forming numerous microcapacitors. These induce strong interfacial polarization under electromagnetic field excitation, further intensifying energy dissipation. The presence of numerous functional groups and structural defects in rGO serves as dipolar polarization centers, contributing to relaxation polarization with a delayed response under electromagnetic excitation and enhancing the overall dielectric loss. Furthermore, the magnetic Fe_3_O_4_ nanoparticles contribute to additional energy dissipation via magnetic loss when subjected to alternating electromagnetic fields. The 3D structure of FCGP‐25 also promotes multiple reflection and scattering events, extending the propagation path of EMW and improving overall attenuation. Therefore, the excellent EMW absorption performance of FCGP‐25 arises from the combined effects of optimized impedance matching and multiple synergistic loss mechanisms (Figure 6c). In this work, these obtained compacting composites clearly exhibit the fundamental attenuation mechanisms, including conduction loss, polarization relaxation, and magnetic resonance, without interference from structural factors. Meanwhile, the structural robustness and stability of obtained products made them potential candidate as raw materials for additively manufacturing, which may exhibit further performances after typically structure, interlayer boundaries, and periodic lattice feature designing.^[^ ^69^, ^70^ ^]^

### 2.5 Mechanical and Thermal Stability Performance

**Figure 7:**
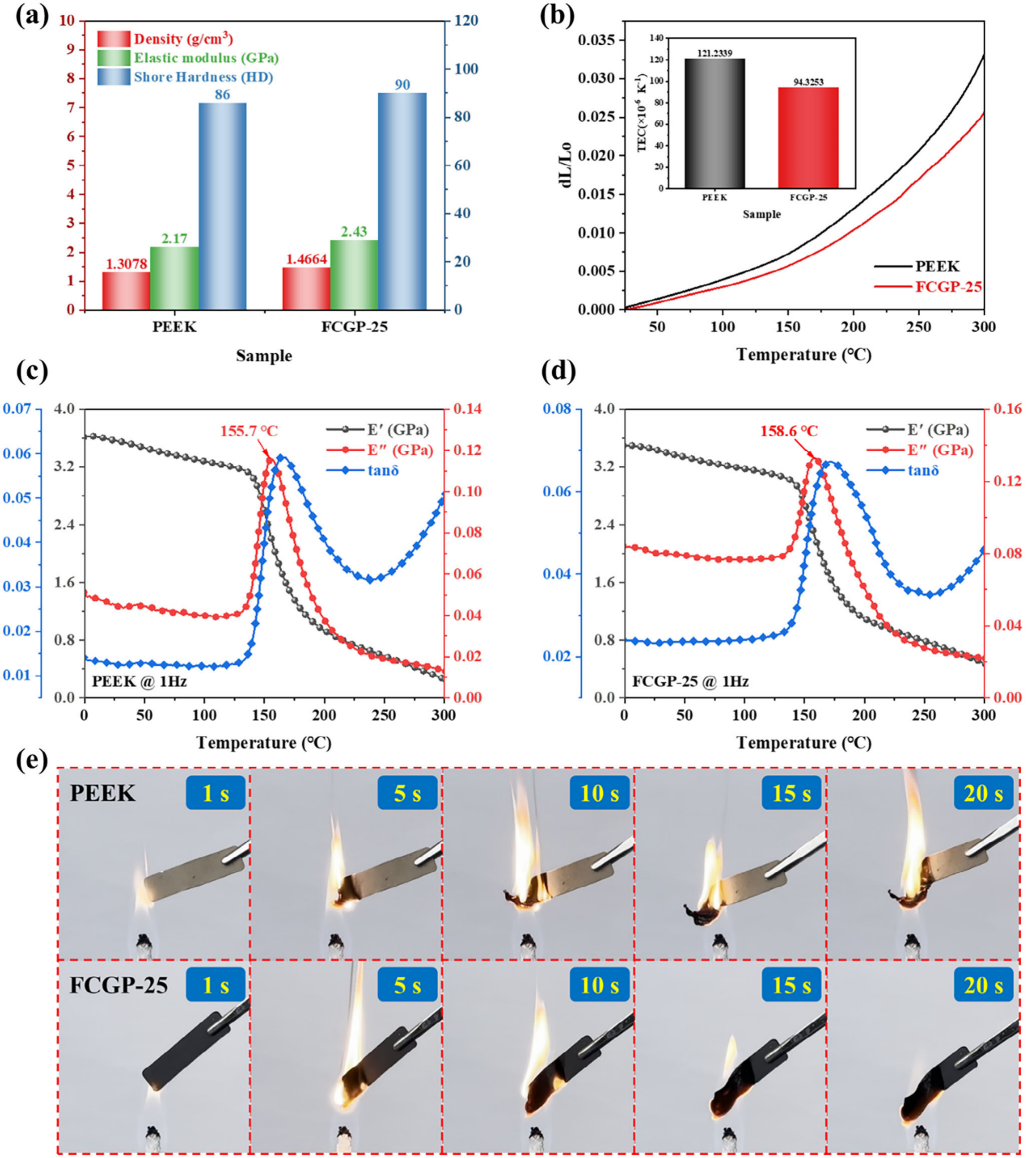
Multifunctional integration of mechanical reliability, thermal stability, and flame retardancy for FCGP‐25. a) Density, elastic modulus, and shore hardness of neat PEEK and FCGP‐25. b) Thermal expansion curves and thermal expansion coefficients (TEC) of neat PEEK and FCGP‐25 in the temperature range of 30–300 °C. c) Dynamic temperature sweep curve and glass transition temperature (*T*_g_) of PEEK at 1 Hz. d) Dynamic temperature sweep curve and *T*_g_ of FCGP‐25 at 1 Hz. e) Combustion process of PEEK and FCGP‐25 under an alcohol lamp.

To systematically evaluate the comprehensive performance of FCGP‐25 comparing to PEEK, a series of multidimensional characterizations were conducted. First, the density of FCGP‐25, measured via the Archimedes drainage method, was found to be 1.46 g cm^−3^, an increase of 12.31% compared to pristine PEEK (1.30 g cm^−3^). This increase is attributed to the incorporation of functional fillers. Nevertheless, the result indicates that the fillers do not significantly compromise the lightweight nature of the PEEK matrix, still in the typical range for engineering plastics. The Shore hardness of FCGP‐25 reaches 90 HD, showing a 4.7% improvement over PEEK (86 HD). To assess the deformability in the small‐strain regime, microtensile tests were carried out using a DMA tensile mode. FCGP‐25 exhibits a higher elastic modulus of 2.43 GPa, compared to 2.17 GPa for the PEEK matrix, indicating that the incorporation of hybrid fillers effectively enhances the stiffness of the PEEK matrix (**Figure** **7**a). Importantly, within the instrument‐limited strain window (up to ≈0.55%), the neat PEEK sample exhibits a maximum stress of about 9 MPa at a strain of 0.5%, while the FCGP‐25 composite shows a slightly higher stress level (up to 11 MPa) and the strain range extends from 0–0.5% to 0–0.55%, indicating that the incorporation of FCG hybrid fillers enhances stiffness without sacrificing ductility (Figure S16, Supporting Information). At a given strain (e.g., 0.45%), FCGP‐25 sustains a higher stress without sacrificing deformability in the service‐relevant small‐strain range. This can be attributed to the uniform dispersion of the FCG fillers and the strong interfacial bonding between the fillers and the polymer matrix, which facilitate efficient stress transfer during deformation. Therefore, the FCGP‐25 composite achieves a balanced improvement in both stiffness and ductility, demonstrating its potential for structural electromagnetic applications where mechanical robustness and flexibility are simultaneously required. Thermal expansion behavior analysis indicates that FCGP‐25 possesses a coefficient of thermal expansion (TEC) of 94.33 × 10^−6^ K^−1^ in the range of 30–300 °C, which is markedly lower than that of PEEK (121.23 × 10^−6^ K^−1^), representing a 22.2% reduction. This result suggests that the filler network effectively suppresses thermally induced deformation of the polymer chains, making the composite more suitable for high‐temperature applications requiring dimensional stability (Figure 7b). Based on DMA dynamic temperature scanning results, the glass transition temperature (*T*_g_) of FCGP‐25 slightly increased from 155.7 to 158.6 °C compared to PEEK. The slightly reduced storage modulus of FCGP‐25 at room temperature arises from interfacial effects between the fillers and polymer chains, which enhance local chain mobility in the amorphous phase. Nevertheless, as temperature increases, the filler reinforcement becomes predominant, leading to an overall improvement in modulus throughout the temperature range. Both the storage modulus (*E′*) and loss modulus (*E″*) of FCGP‐25 demonstrate a smoother transition in the high‐temperature region, particularly near *T*_g_, indicating improved mechanical performance and thermodynamic stability relative to PEEK. The tan*δ* curve also exhibits a flatter and lower peak around *T*_g_, suggesting reduced mechanical damping at elevated temperatures. These observations further confirm the positive influence of the filler on enhancing both thermal and mechanical properties, enabling the composite to maintain structural integrity under more demanding thermal conditions (Figure 7c,d). During the flame retardancy test, the PEEK sample sustained combustion under an alcohol lamp flame for more than 20 s and exhibited significant curling deformation. By contrast, FCGP‐25 demonstrated a rapid self‐extinguishing characteristic, with a burning time of ≈20 s (Figure 7e). This phenomenon indicates that the FCGP composite significantly enhances the flame retardancy of the material. These multiscale characterization results demonstrate that FCGP‐25 achieves simultaneous enhancements in mechanical strength, thermal stability, and flame retardancy while maintaining lightweight characteristics. These advantages highlight its strong potential for advanced applications in aerospace and high‐temperature stealth technologies.

## 3 Conclusion

In conclusion, the high‐performance PEEK‐based composites with efficient EMW absorption with broad temperature adaptability have been prepared through a facile and flexible strategy. The ternary nanofiller system was selected to activate multiple complementary loss mechanisms: Fe_3_O_4_ contributes magnetic loss via natural resonance and eddy current dissipation; CNTs offer excellent electrical conductivity, supporting conduction loss and enhancing interfacial polarization; rGO introduces additional interfaces and electric dipoles, further amplifying dielectric loss while improving filler dispersion. The synergy among these components, coupled with the high thermal resistance and mechanical integrity of the PEEK matrix, not only provides the composites a stable structure, but also the excellent EMW absorption performances across 298–573 K. Experimental results demonstrate that the optimized composite achieves a minimum reflection loss of −66.62 dB and a maximum effective absorption bandwidth of 4.58 GHz at room temperature. Notably, these outstanding absorption characteristics are well retained at 573 K (RL_min_ = −66.01 dB, EAB_max_ = 4.05 GHz), highlighting excellent thermal robustness. Beyond absorption efficiency, the composite also exhibits improved thermal stability and mechanical strength compared to pristine PEEK, indicating its applicability in structurally demanding environments. The composite's broad‐band, temperature‐tolerant absorption performance surpasses most previously reported thermoplastic systems. Furthermore, the compatibility of PEEK with additive manufacturing and other scalable processing techniques enables the precise construction of complex geometries, paving the way for future applications in multifunctional structural–electromagnetic integration. Overall, this work not only establishes a feasible approach to constructing wide‐temperature‐range EMW absorbing thermoplastic composites, but also provides fundamental insights into the filler–matrix–structure interaction mechanisms essential for designing next‐generation electromagnetic protection materials operating in a wide temperature range.

## 4 Experimental Section

The main experimental procedures for the FCGP series of materials included the synthesis of FC, FG, and FCG nanomaterials by a synthetic solvent thermal method to obtain magnetic carbon nanomaterials followed by mechanical mixing with PEEK powders. Detailed experimental process is available in the Supporting Information.

## Conflict of Interest

The authors declare no conflict of interest.

## Supporting information



Supporting Information

## Data Availability

The data that support the findings of this study are available from the corresponding author upon reasonable request.
