# Supplementary material for: Facile Manufacturing of PEEK‐Based Nanocomposites for High‐Efficiency Wide‐Temperature‐Range Electromagnetic Wave Absorption
Source: Adv Sci (Weinh). 2025 Nov 26;13(8):e23051. doi: 10.1002/advs.202523051 (PMC12884812; doi:10.1002/advs.202523051)
Supplement: Supplementary file 1 — Supporting Information [file ADVS-13-e23051-s001.docx]

**Supporting Information**

**Facile Manufacturing of PEEK-Based Nanocomposites for High-Efficiency Wide-Temperature-Range Electromagnetic Wave Absorption**

Liang Zhao, Jingpeng Lin, Zifan Zheng, Yajun Chen, Xiong Yang, Yu Han, Yuchan Cheng*, Chenxi Hu*, Zhihui Zeng*

L. Zhao, Y. Chen, C. Hu

Sino-European Institute of Aviation Engineering

Civil Aviation University of China

Tianjin 300300, China

E-mail: cxhu@cauc.edu.cn

J. Lin, Z. Zeng

Key Laboratory for Liquid−Solid Structural Evolution and Processing of Materials (Ministry of Education), School of Materials Science and Engineering

Shandong University

Jinan 250061, China

E-mail: zhihui.zeng@sdu.edu.cn

Z. Zheng, X. Yang, C. Hu

College of Science

Civil Aviation University of China

Tianjin 300300, China

E-mail: cxhu@cauc.edu.cn

Y. Han

Tianjin Istar-Space Technology Co., Ltd.

Tianjin 300300, China

Y. Cheng

Laboratory of Atomic-Scale and Micro & Nano Manufacturing

Ningbo Institute of Materials Technology & Engineering, Chinese Academy of Sciences

Ningbo 315201, China

E-mail: yccheng@nimte.ac.cn

**1. Experimental details**

***Materials:*** The graphene oxide (GO) slurry and carbon nanotubes (CNTs) used in this study were purchased from Changzhou Angxing Advanced Carbon Materials Co., Ltd., China. Triethylene glycol (TEG) and ferric nitrate nonahydrate (Fe(NO_3_)_3_·9H_2_O) were obtained from Macklin Biochemical Technology Co., Ltd., Shanghai, China, while PEEK was provided by Victrex, UK.

***Synthesis of Fe_3_O_4_/CNTs/rGO nanocomposites:*** The nanocomposite was synthesized via a solvothermal method. Specifically, 10 mg of graphene oxide (GO) and 10 mg of carbon nanotubes (CNTs) were separately dispersed in 20 mL of triethylene glycol (TEG) and ultrasonicated for 15 minutes to obtain homogeneous dispersions, denoted as Solution A and Solution B, respectively. Subsequently, these two solutions were combined and further ultrasonicated for another 15 minutes, forming a uniform Solution C. Meanwhile, 0.202 g of Fe(NO_3_)_3_·9H_2_O was completely dissolved in 20 mL of TEG under magnetic stirring for 15 minutes until a homogeneous orange-transparent solution was obtained. Solution C was then mixed with the ferric nitrate solution, followed by another 15 minutes of magnetic stirring. The resulting mixture was transferred into a stainless-steel autoclave with a Teflon liner and subjected to a solvothermal reaction at 180 °C for 4 hours. After the reaction, the product was naturally cooled to room temperature, collected via centrifugation, and thoroughly washed with deionized water and ethanol for purification. Finally, the purified product was dried at 70 °C to obtain Fe_3_O_4_/CNTs/rGO nanocomposites, which are referred to as FCG in the following sections. Similarly, nanocomposites without CNT or GO were named FC and FG, respectively.

***Fabrication of*** ***Fe_3_O_4_/CNTs/rGO/PEEK composites and comparison sample:*** First, a certain amount of FCG and PEEK powders were weighed, mixed in predetermined ratios, and thoroughly ground to obtain three composites with different FCG mass fractions of 20%, 25%, and 30%. For simplicity, these composites are denoted as FCGP-X, where X represents the mass ratio of FCG in the composite. Similarly, the composites prepared by mixing Fe_3_O_4_, FC or FG with PEEK are named FP-X, FCP-X or FGP-X, respectively.

***Characterization:*** Cu K-α radiation was used as the X-ray source for X-ray diffraction (XRD; Bruker D8 ADVANCE, Germany) analysis of the sample’s crystal structure within the scanning range of 10<2θ<80°, with a working voltage and current of 40 kV and 40 mA, respectively. The scan step size was set at 0.02°, with a test speed of 0.1 s per step. X-ray photoelectron spectroscopy (XPS, Thermo ESCALAB 250XI) was used to analyze the elemental composition and chemical states of the sample, with Al K-α X-rays (E = 1486.68 eV) as the excitation source and a working voltage of 12.0 kV. The sample surface was scanned using a field emission scanning electron microscope (FESEM; Zeiss Sigma 360, Germany) and a high-resolution transmission electron microscope (HRTEM; FEI Tecnai G2 F30, USA), obtaining high-resolution images to observe the morphology, size, and distribution of the sample. The hysteresis loop of the sample was measured using a superconducting quantum interference magnetic measurement system (MPMS, Quantum Design MPMS-XL-7, USA) at room temperature. Thermogravimetric analysis (TGA, NETZSCH TG 209 F3, Germany) was performed in an air atmosphere to obtain the thermogravimetric curve, with a testing temperature range from room temperature to 900 °C and a heating rate of 10 °C/min. The thermal expansion coefficient (TEC) was measured using a dilatometer (NETZSCH DIL 402 Expedis Classic, Germany) in the temperature range of 30-300 °C with a heating rate of 5 °C/min. The mechanical properties of the materials were characterized using a dynamic mechanical analyzer (DMA, HITACHI DMA200), with dynamic temperature scanning and stress-strain modes applied to determine the glass transition temperature and Young’s modulus, within the temperature range of 10-300 °C, a heating rate of 2 °C/min, and a test frequency of 1 Hz. A vector network analyzer (VNA, Ceyear 3672B, China) was used to measure the electromagnetic parameters of the material in the frequency range of 2-18 GHz using the coaxial method. Briefly, the prepared FCGP-X powders were pressed into custom-designed metal molds under a pressure of 5 MPa to form toroidal samples with an outer diameter of 7.00 mm and an inner diameter of 3.04 mm. In addition, the composites were machined into rectangular samples with dimensions of 22.86×10.16×2 mm^3^ for the characterization of their electromagnetic properties in the 8.2–12.4 GHz (X-band) range using the wave-guide method.

**2. Static magnetic properties and theoretical calculations of EMW absorption performance.**

***2.1 Ferromagnetic resonance theory***

An anisotropic field exists within the material due to the ferromagnetic nature of the material, meaning that a resonance phenomenon known as natural resonance occurs internally even in the absence of an external magnetic field. Ferromagnetic resonance theory provides a relationship between the natural resonance frequency and the anisotropic field: the lower the *H_c_* value, the better absorption performance in the mid/low-frequency range could be found. This phenomenon can be explained by the following formula:^[1]^

$2\pi f_{r}=\gamma H_{a}$ (1)

$H_{a}=4|K_{1}|/3\mu_{0}M_{s}$ (2)

$K_{1}=\mu_{0}M_{s}H_{c}/2$ (3)

In the given Equation (1), (2) and (3), *f_r_* denotes the resonance frequency, *γ* represents the gyromagnetic ratio, *H_a_* correspond to the anisotropy energy, *K*_1_ is the anisotropy constant, and *μ*_0_ denotes the permeability of free space. A straightforward mathematical derivation demonstrates that the coercivity *H_c_* is directly proportional to the resonance frequency *f_r_*.

Magnetic loss is another important factor of the absorption mechanism in EMW absorbers. The primary magnetic loss mechanisms include eddy current loss, natural resonance, exchange resonance, hysteresis loss, and domain wall loss, each corresponding to a resonance peak in the *μ''*-*f* curve. In the 2-18 GHz frequency range, only the first three mechanisms are taken into account, as hysteresis loss can be ignored in weak electromagnetic fields, and domain wall loss typically occurs in the MHz region.^[2]^ Natural resonance refers to the resonance of intrinsic or magnetic dipoles within the material when subjected to an external electromagnetic field, typically causing dispersion and energy dissipation in the dielectric constant or permeability. This phenomenon generally occurs in the low-frequency range (2–10 GHz) and depends on the material's size, shape, and composition.^[1c, 3]^

***2.2 Reflection loss (RL)***

Reflection loss (RL) is used to reflect the wave-absorbing performance of materials. According to transmission line theory, its calculation formula is as follows:

$RL\left(\text{dB}\right)=20\log\left|\frac{Z_{in}-Z_{0}}{Z_{in}+Z_{0}}\right|$ (4)

$Z_{in}=Z_{0}\sqrt{\frac{\mu_{r}}{\varepsilon_{r}}}\tanh\left(j\frac{2\pi fd}{c}\sqrt{\mu_{r}\varepsilon_{r}}\right)$ (5)

In the given Equation (4) and (5), *Z_in_* is the input impedance of the material, *Z*_0_ is the impedance of free space (*Z*_0_ = 376.7 Ω), *f* is the frequency of the EMW, *d* is the thickness of the material, *c* is the speed of light in a vacuum, and *j* is the imaginary unit. When the absolute value of the reflection loss is greater than 10 dB (i.e., RL ≤ −10 dB), more than 90% of the EMW are absorbed, which is called the effective absorption of EMW. Similarly, a reflection loss of −20 dB indicates that 99% of the EMW are absorbed, and so on. The total frequency range that a material can effectively absorb EMW is called the effective absorption bandwidth (EAB).^[4]^ The larger the value of EAB, the wider the frequency range over which the material can effectively absorb EMW.

***2.3 Impedance matching coefficient.***

To better explain the differences in performance, impedance matching was introduced to evaluate the EMW entering performance of the samples, and the calculation formula is as Equation (6):^[5]^

$Z=\left|\frac{Z_{in}}{Z_{0}}\right|$ (6)

When *Z* equals 1, EMW can fully enter the material without reflection, achieving ideal impedance matching. It is generally considered that materials exhibit good impedance matching when Z is in the range of 0.8 to 1.2.^[6]^

***2.4 Cole-Cole plot and Debye relaxation theory.***

The Cole-Cole plot, based on Debye relaxation theory, can be used to analyze the polarization relaxation process. The relationship between *ε'* and *ε''* can be expressed as follows:

$\left(\varepsilon'-\frac{\varepsilon_{s}+\varepsilon_{\infty}}{2}\right)^{2}+\left(\varepsilon''\right)^{2}=\left(\frac{\varepsilon_{s}+\varepsilon_{\infty}}{2}\right)^{2}$ (7)

In the given Equation (7), *ε_s_* and *ε_∞_* represent the static permittivity at frequencies approaching 0 and the limiting permittivity at frequencies approaching infinity, respectively. The number of semicircles in the plot indicates the strength of polarization losses, while the straight line at the end of the curve represents the conductive loss process, with the slope indicating the intensity of the conductive loss.^[7]^ polarization loss and conductive loss with frequency, Equation (8), (9) and (10) are ultilised to further study the both EMW loss types for FCGP-25:

$\varepsilon''=\varepsilon''_{p}+\varepsilon''_{c}=(\varepsilon_{s}-\varepsilon_{\infty})\frac{2\pi f\tau}{1+(2\pi f)^{2}\tau^{2}}+\frac{\sigma}{2\pi f\varepsilon_{0}}$ (8)

$\varepsilon''_{c}=\frac{\sigma}{2\pi f\varepsilon_{0}}$ (9)

$\varepsilon''_{p}=(\varepsilon_{s}-\varepsilon_{\infty})\frac{2\pi f\tau}{1+\left(2\pi f\right)^{2}\tau^{2}}$ (10)

Here, $\varepsilon''_{p}$ represents polarization loss, $\varepsilon''_{c}$represents conductive loss, *σ* is the material’s conductivity, and *ε*_0_ (*ε*_0_ = 8.854 × 10^−12^ F/m) is the permittivity of free space.

***2.5 C_0_ value.***

According to ferromagnetic resonance theory, the eddy current loss coefficient *C*_0_ is directly related to the real part (*μ′*), imaginary part (*μ′′*) of the complex permeability, and frequency (*f*), expressed as the following Equation (11):

$C_{0}=\mu''(\mu')^{-2}f^{-1}=2\pi\mu_{0}d^{2}\sigma/3$ (11)

where *μ*_0_ = 4π × 10^−7^ H/m is the permeability of free space, *d* is the material thickness, and *σ* is the material conductivity (S/m).^[8]^ This formula introduces the complex permeability parameters, linking the microscopic mechanism of eddy current loss with the macroscopic electromagnetic response. Since the conductivity is constant, *C*_0_ should remain constant when the material thickness is fixed, indicating that when the magnetic loss mechanism is completely resulted from eddy current loss, *C*_0_ does not vary with frequency, appearing as a straight line on the *C*_0_-*f* curve.

***2.6 Attenuation coefficient (α).***

The attenuation coefficient (*α*) is a key parameter for evaluating the overall EMW attenuation capability of materials, defined as:

$\alpha=\frac{\sqrt{2}\pi f}{c}\sqrt{(\mu''\varepsilon''-\mu'\varepsilon')+\sqrt{(\mu''\varepsilon''-\mu'\varepsilon')^{2}+(\mu'\varepsilon''+\mu''\varepsilon')^{2}}}$ (12)

In the given Equation (12), *f* is the frequency of the incident EMW, and c is the speed of EMW in vacuum.^[9]^ By analyzing the variation of *α* with frequency *f*, the EMW absorption mechanism of the material can be revealed. The larger the *α* value, the stronger the EMW attenuation ability. Specifically, the peaks in the curve correspond to the material's optimal absorption frequency, indicating that dielectric loss (*ε′′*) and magnetic loss (*μ′′*) could effectively collaborate at this frequency,. The steep increase in the low-frequency region may be attributed to dielectric losses caused by interface polarization, while the peaks in the mid-frequency region are due to resonance losses (e.g., dipole resonance, natural resonance, exchange resonance, etc.). The slowing of the curve in the high-frequency region is due to the effects of eddy current losses and multiple relaxation processes.

***2.7 Quarter-wavelength theory.***

In addition to the interactions between the components of the material itself, the quarter-wavelength theory (*λ*/4 theory) is also a key model for optimizing the performance of EMW absorption materials. The core principle involves adjusting the material thickness (*t_m_*) to achieve phase cancellation of the reflected wave, thus enhancing microwave absorption. Based on the principle of interference cancellation, the thickness of the material should satisfy the following relationship with the wavelength of the EMW:

$t_{m}=n\lambda/4=nc/\left(4f_{m}\sqrt{|\varepsilon_{r}||\mu_{r}|}\right) (n=1,3,5\ldots)$ (13)

In the given Equation (13), *t_m_* represents the matching thickness, *λ* = *c*/*f_m_* is the wavelength in vacuum, *c* is the speed of light (3 × 10^8^ m/s), and *f_m_* is the optimal absorption frequency corresponding to the matching thickness. $|\varepsilon_{r}|=\sqrt{(\varepsilon')^{2}+(\varepsilon'')^{2}}$and $|\mu_{r}|=\sqrt{(\mu')^{2}+(\mu'')^{2}}$ represent the relative complex permittivity and complex permeability modulus, respectively.^[10]^ This formula indicates that the optimal thickness is jointly determined by the material's electromagnetic parameters and frequency. The reflected EMW that returns to the material surface has the same amplitude as the incident wave but a phase difference of 180°, resulting in interference cancellation and EMW attenuation.

**3. RCS simulation by CST Microwave studio.**

The radar cross section (RCS) is an important parameter for measuring the reflection ability of an object to EMW, quantifying the ratio of reflected EMW intensity to incident wave intensity under radar illumination. The larger the RCS value, the stronger the radar wave reflection and the more easily detectable the object is. Conversely, the smaller the RCS value, the better the stealth effect. RCS is influenced not only by the target's size, shape, and material, but also by factors such as the incident angle and frequency of the EMW.^[11]^

**4. Temperature-dependent complex permittivity.**

$\tau\left(\text{T}\right)=\tau_{0}\exp\left(\text{E}_{\text{a}}\text{/RT}\right)$ (14)

$\varepsilon'=\varepsilon_{\infty}+\frac{\varepsilon_{s}-\varepsilon_{\infty}}{1+\left(2\pi f\right)^{2}\tau\left(\text{T}\right)^{2}}$ (15)

According to the Arrhenius equation (Equation (14)), *τ*, *τ_0_*, E_a_, and T correspond to the relaxation time, pre-exponential factor, activation energy, and temperature, respectively. ^[12]^Consistent with Equation (7), *ε_s_* and *ε_∞_* denote the static permittivity and high-frequency limit permittivity. A simplified derivation combining Equation (8), (14), and (15) reveals a positive correlation between *ε′*, *ε′′* and T, indicating that at a constant frequency, the permittivity increases with temperature.

**5. Supplementary Figures**

**
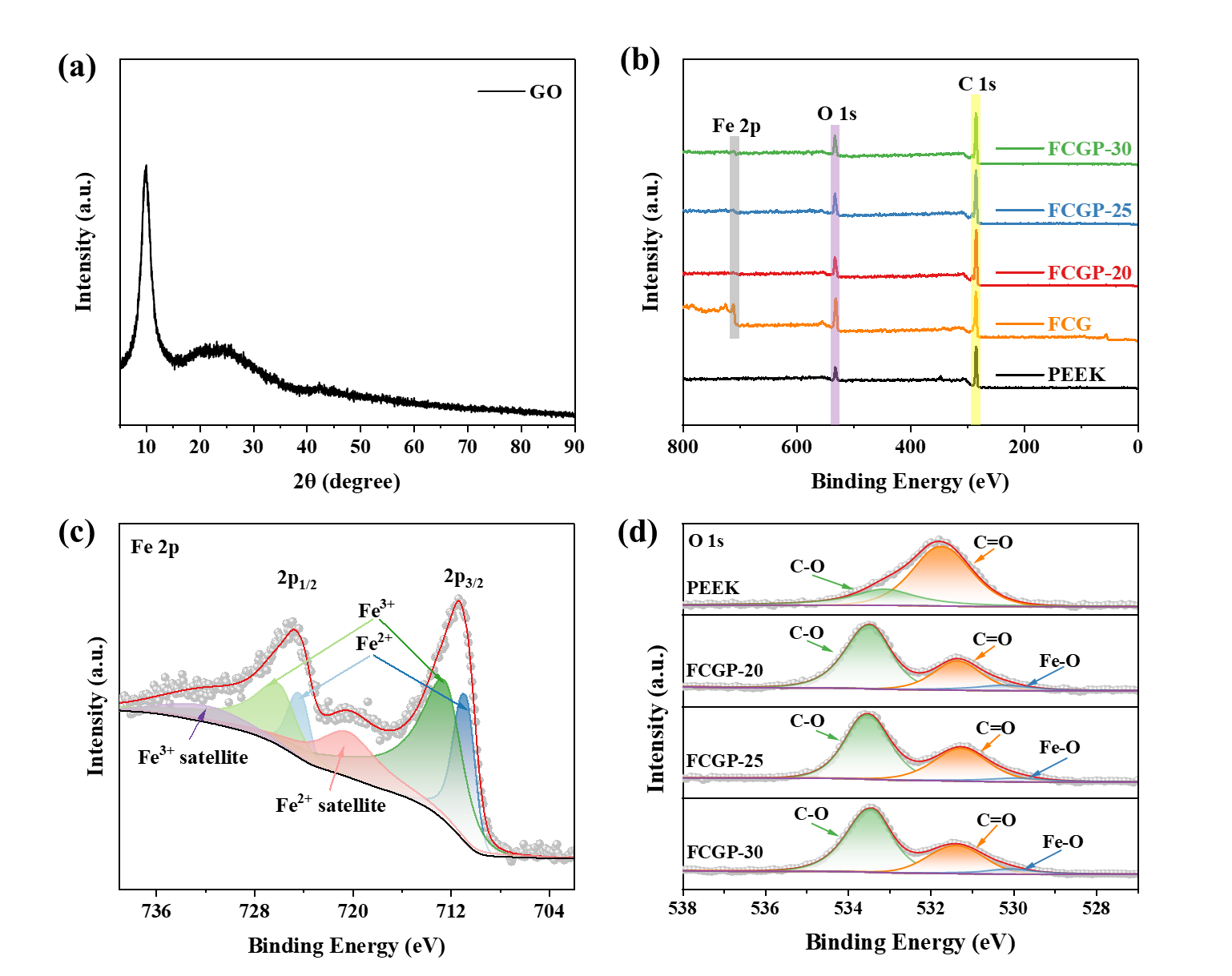
**

**Figure S1.** Structural and Chemical Characterization. a) XRD pattern of GO. b) Survey XPS spectra of FCG nanomaterials, FCGP composites, and PEEK. c) XPS spectrum of the Fe 2p region for FCG nanomaterials. d) XPS spectrum of the O 1s region for PEEK and FCGP composites.

**
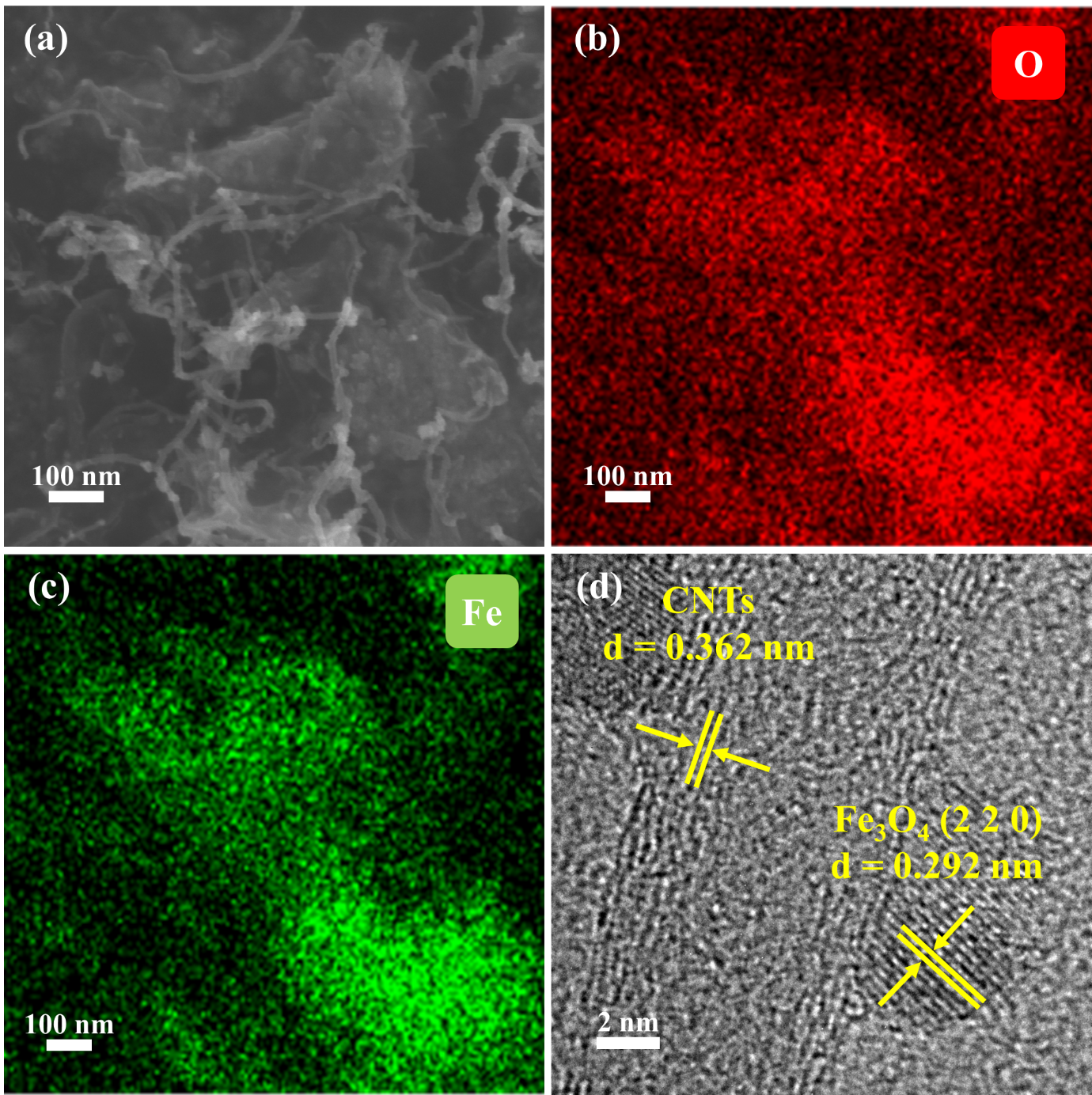
**

**Figure S2.** Morphological and Elemental Characterization of FCG Nanomaterials. a) SEM images of FCG nanomaterials. b)-c) Elemental mapping of O and Fe in FCG nanomaterials. d) TEM images of FCG nanomaterials.


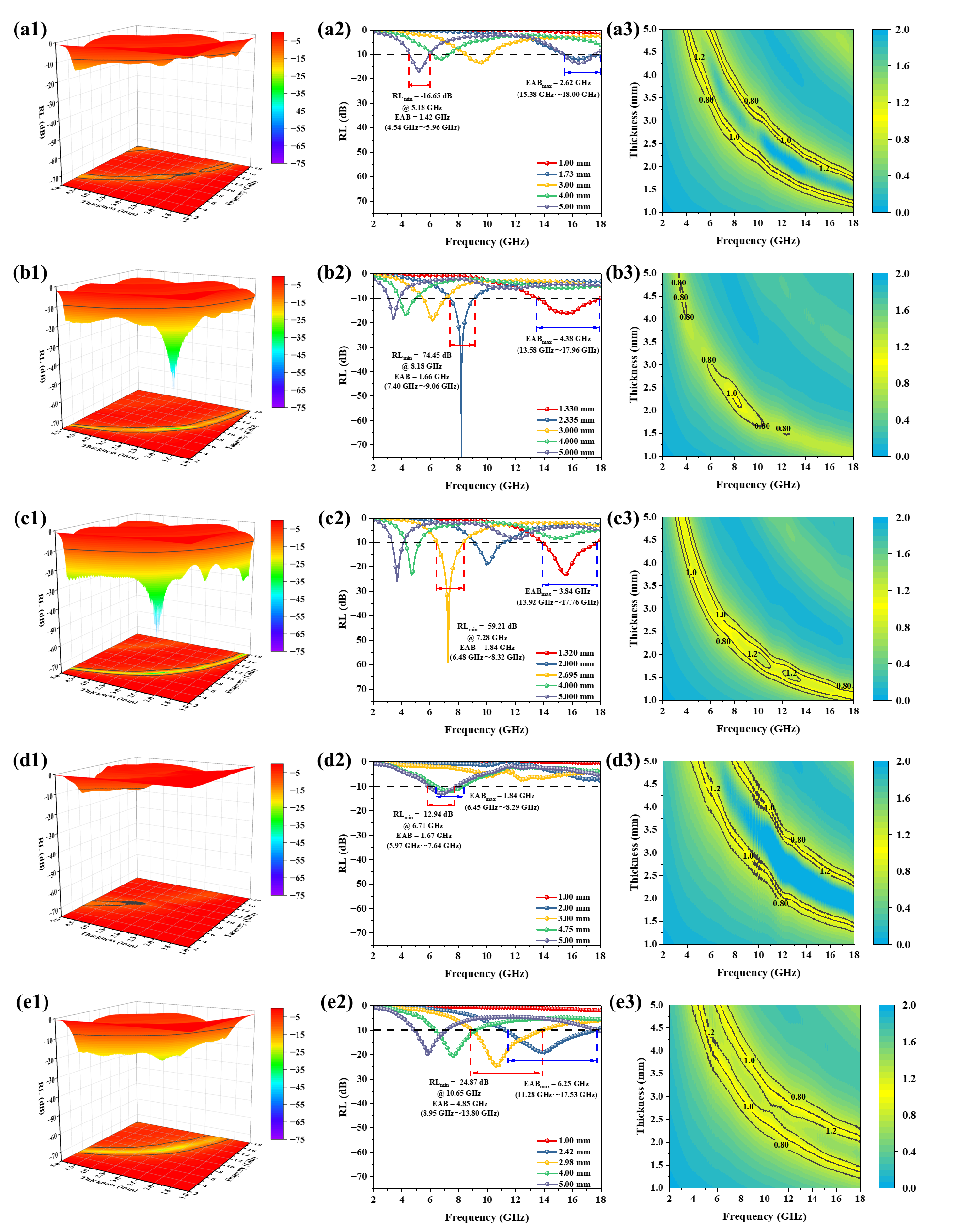


**Figure S3. Reflection Loss and Impedance Matching of Various Composites**

(a1-a3) 3D RL curves, 2D RL curves, and impedance matching of FCGP-20.

(b1-b3) 3D RL curves, 2D RL curves, and impedance matching of FCGP-30.

(c1-c3) 3D RL curves, 2D RL curves, and impedance matching of FCP-25.

(d1-d3) 3D RL curves, 2D RL curves, and impedance matching of FGP-25.

(e1-e3) 3D RL curves, 2D RL curves, and impedance matching of FP-25.


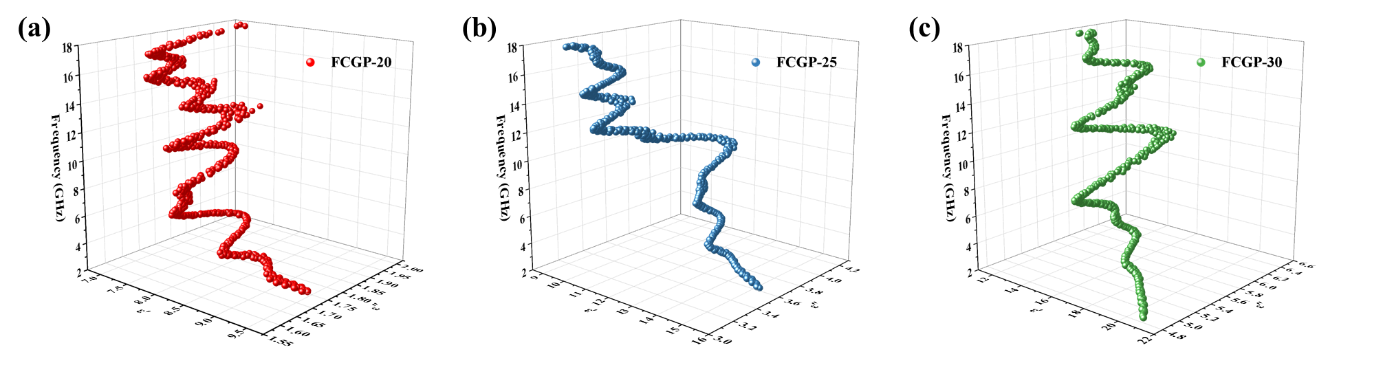


**Figure S4.** 3D Cole-Cole Plots of a) FCGP-20. b) FCGP-25. c) FCGP-30.


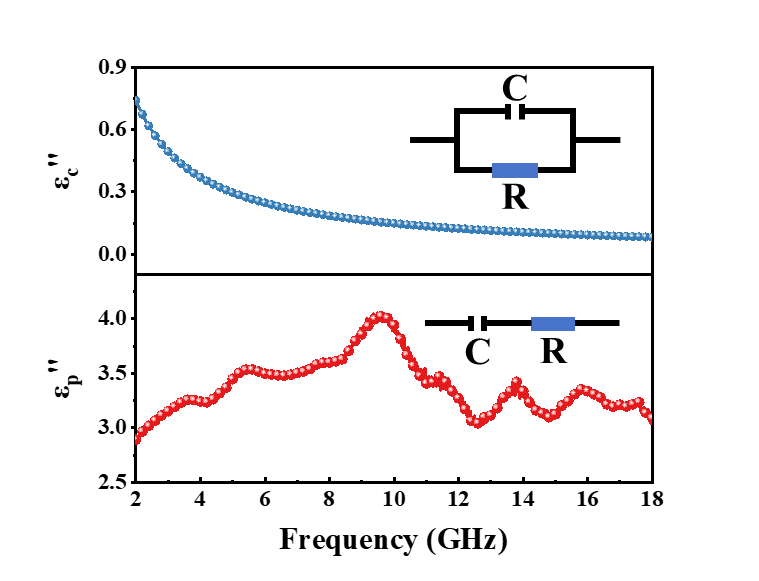


**Figure S5.** Variation of conductivity loss and polarization loss with frequency for FCGP-25.


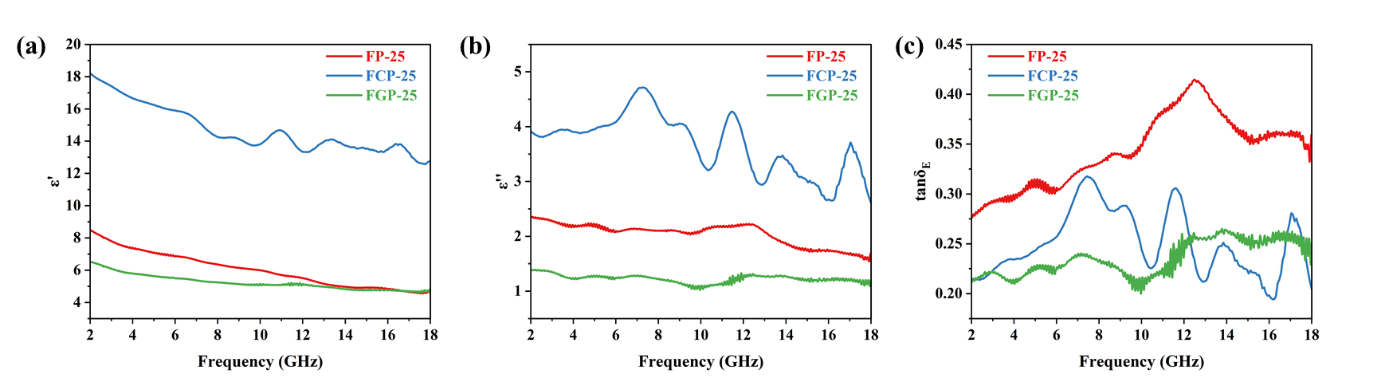


**Figure S6.** a) The real part (*ε′*), b) imaginary part (*ε′′*) of permittivity and c) dielectric loss tangent (tan*δ_E_*) of FP-25, FCP-25 and FGP-25 composites.


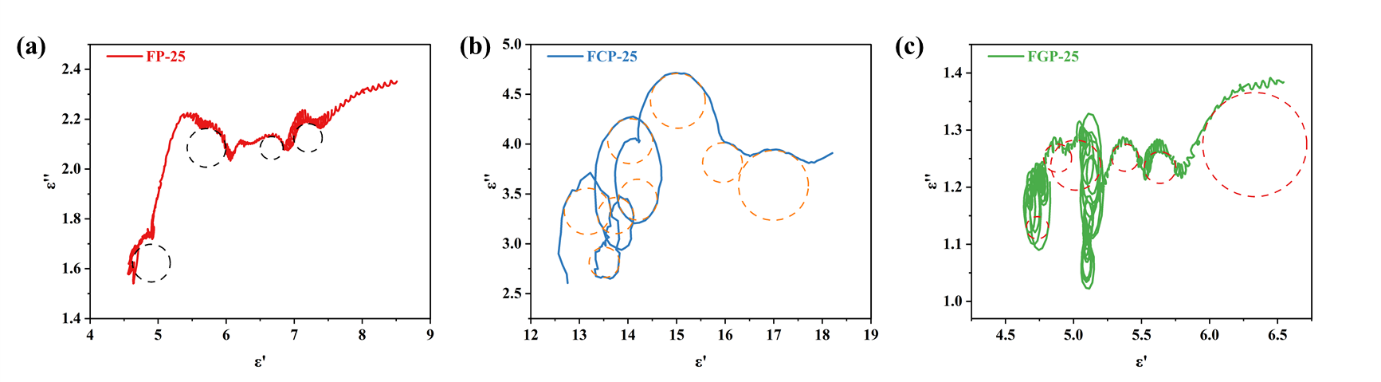


**Figure S7.** Cole-Cole plots of a) FP-25, b) FCP-25 and c) FGP-25 composites.


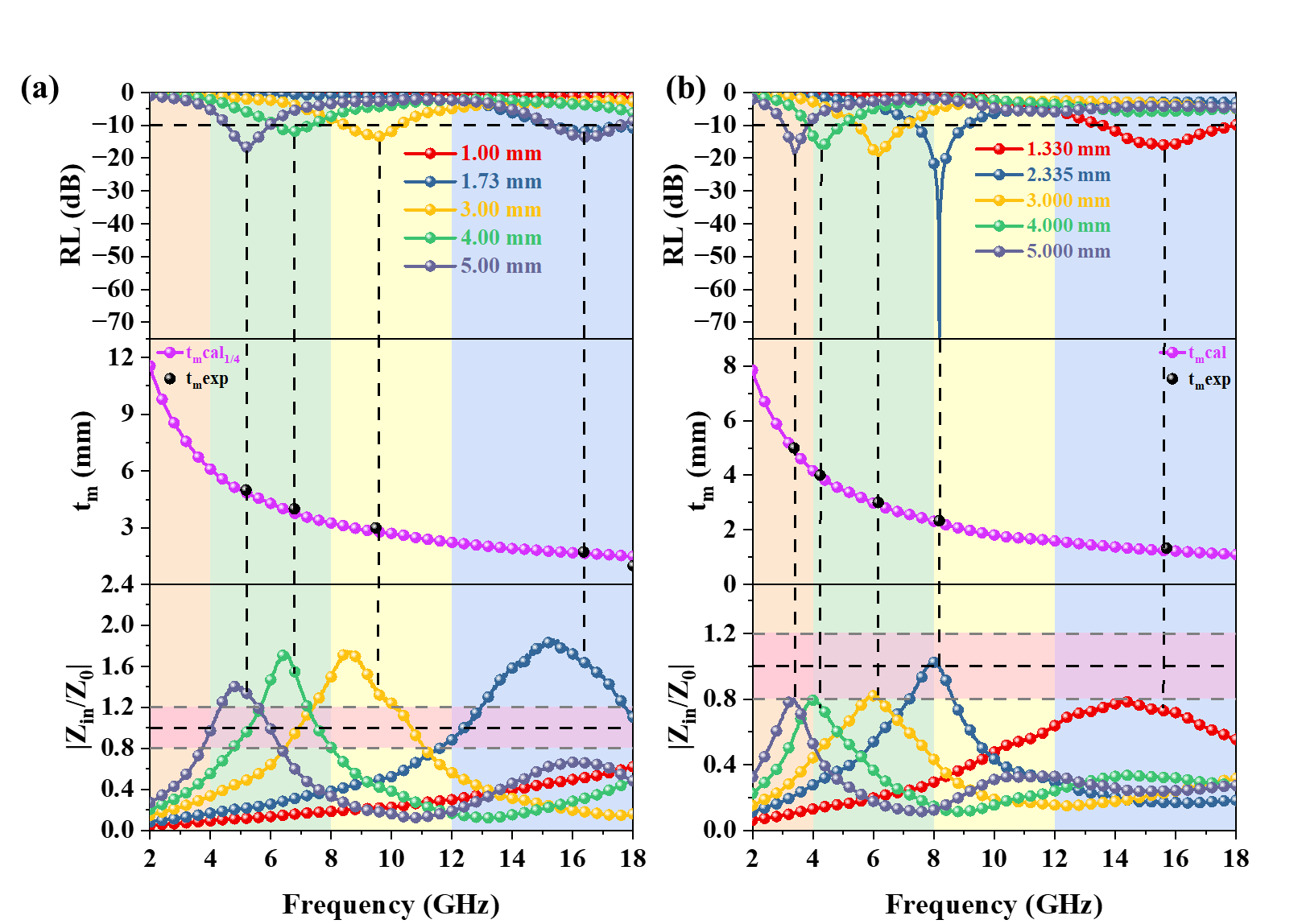


**Figure S8.** 2D RL curves, quarter-wavelength (λ/4), and impedance matching for a) FCGP-20. b) FCGP-30.


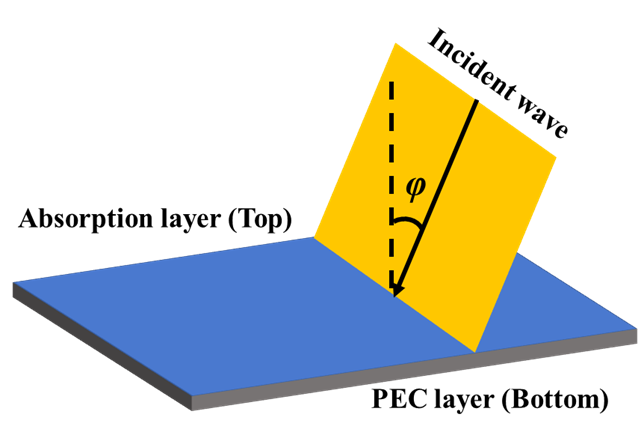


**Figure S9.** PEC model in RCS simulation.


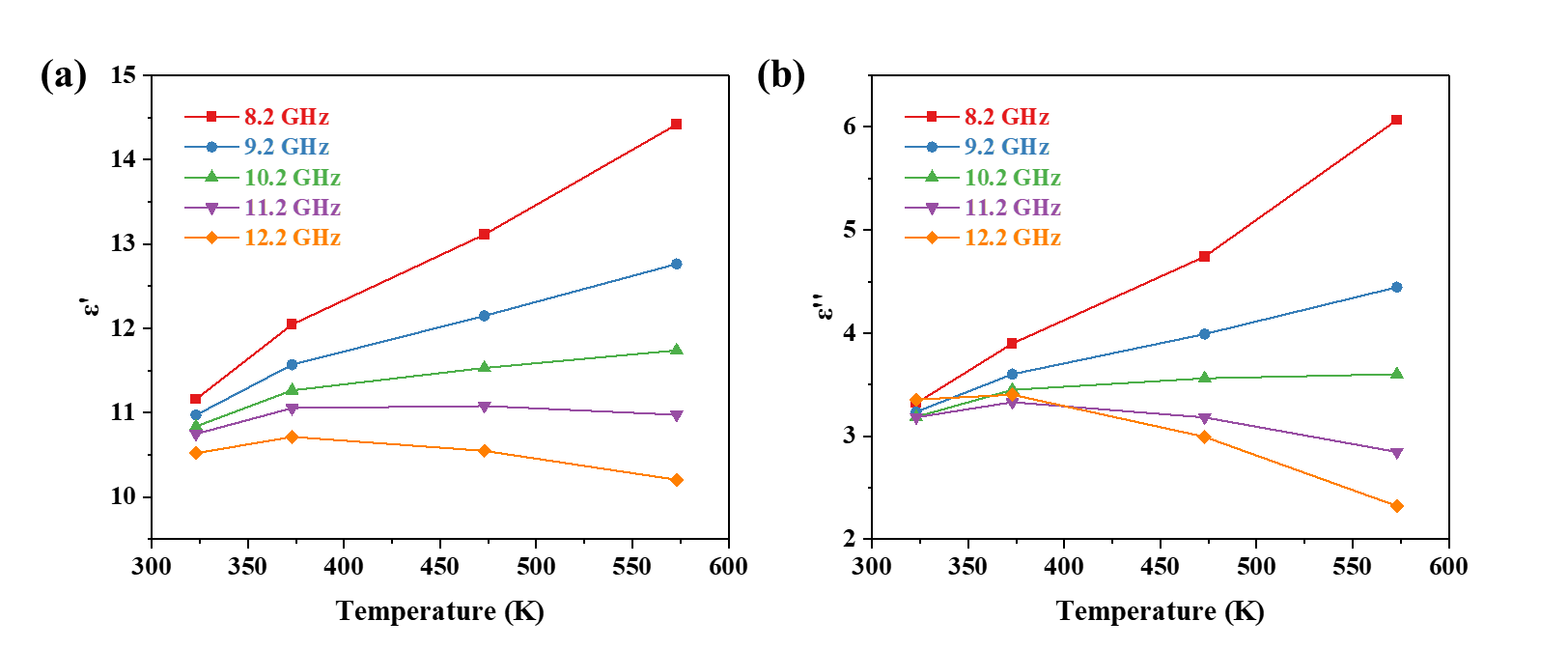


**Figure S10.** a) Temperature-dependent real part (*ε′*) of the complex permittivity of FCGP-25. b) Temperature-dependent imaginary part (*ε′′*) of the complex permittivity.


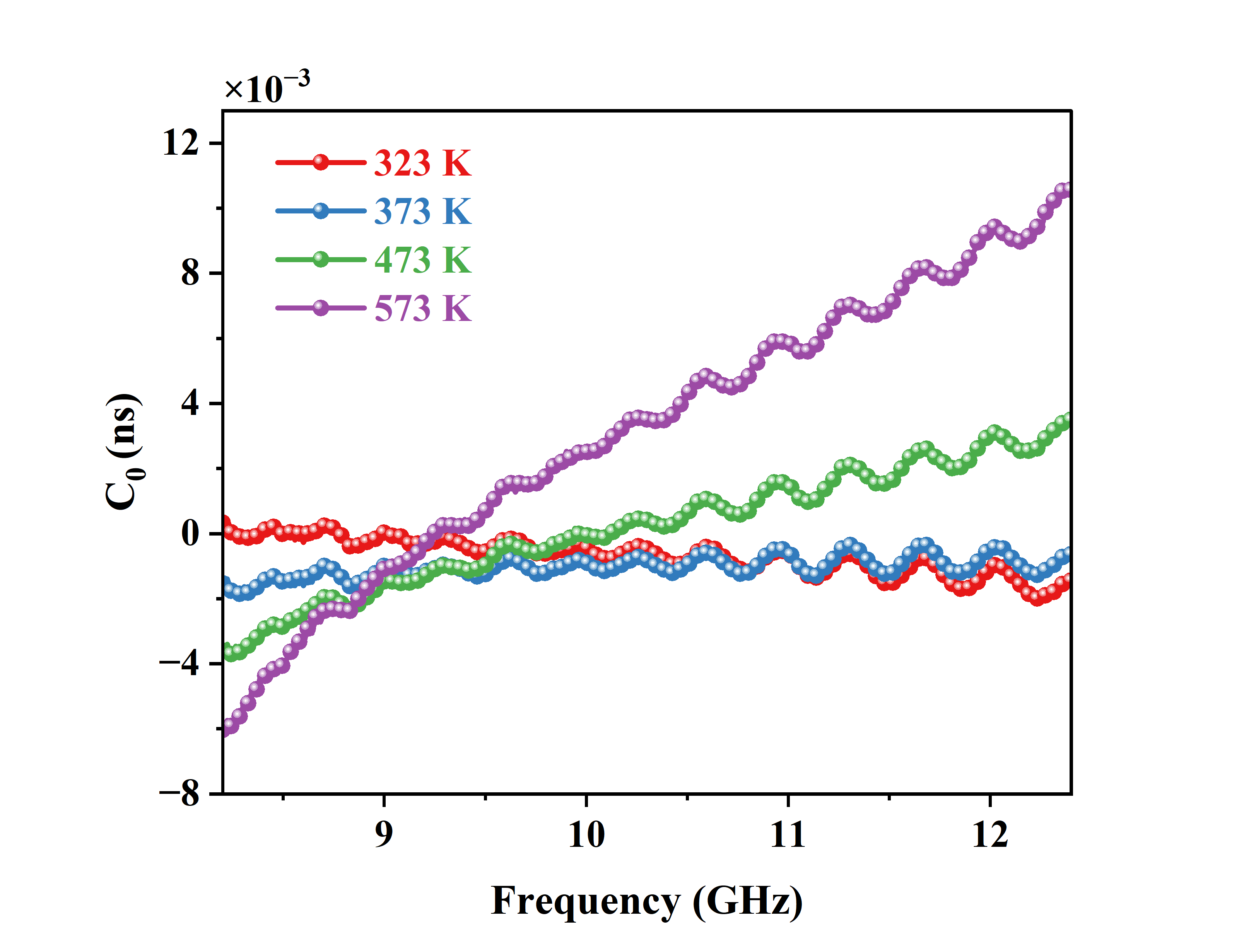


**Figure S11.** Eddy current loss (*C*_0_–*f*) curves at different temperatures.

**
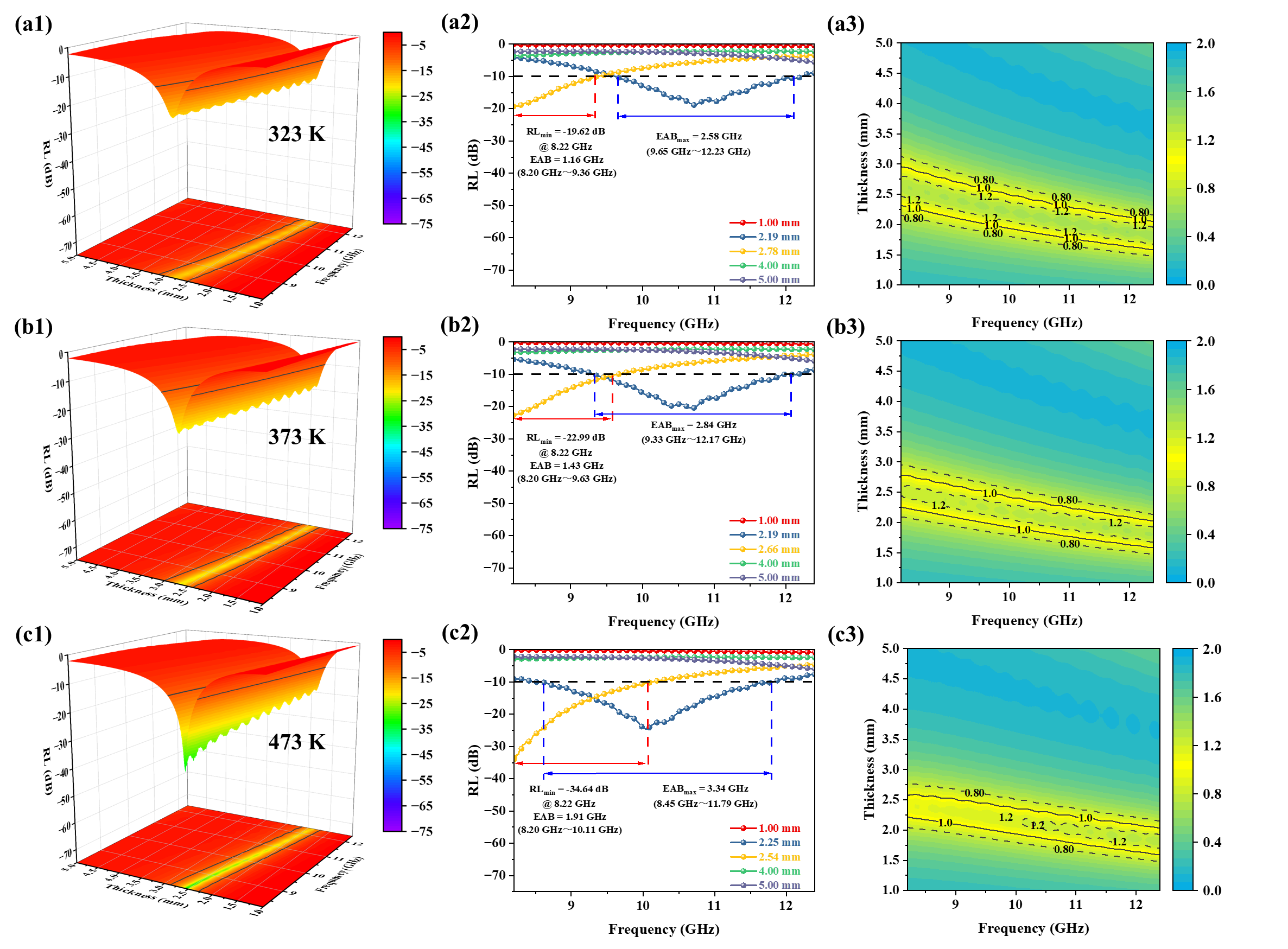
**

**Figure S12.** (a1-a3) 3D RL curves, 2D RL curves, and impedance matching of FCGP-25 at 323 K. (b1-b3) 3D RL curves, 2D RL curves, and impedance matching of FCGP-25 at 373 K. (c1-c3) 3D RL curves, 2D RL curves, and impedance matching of FCGP-25 at 473 K.


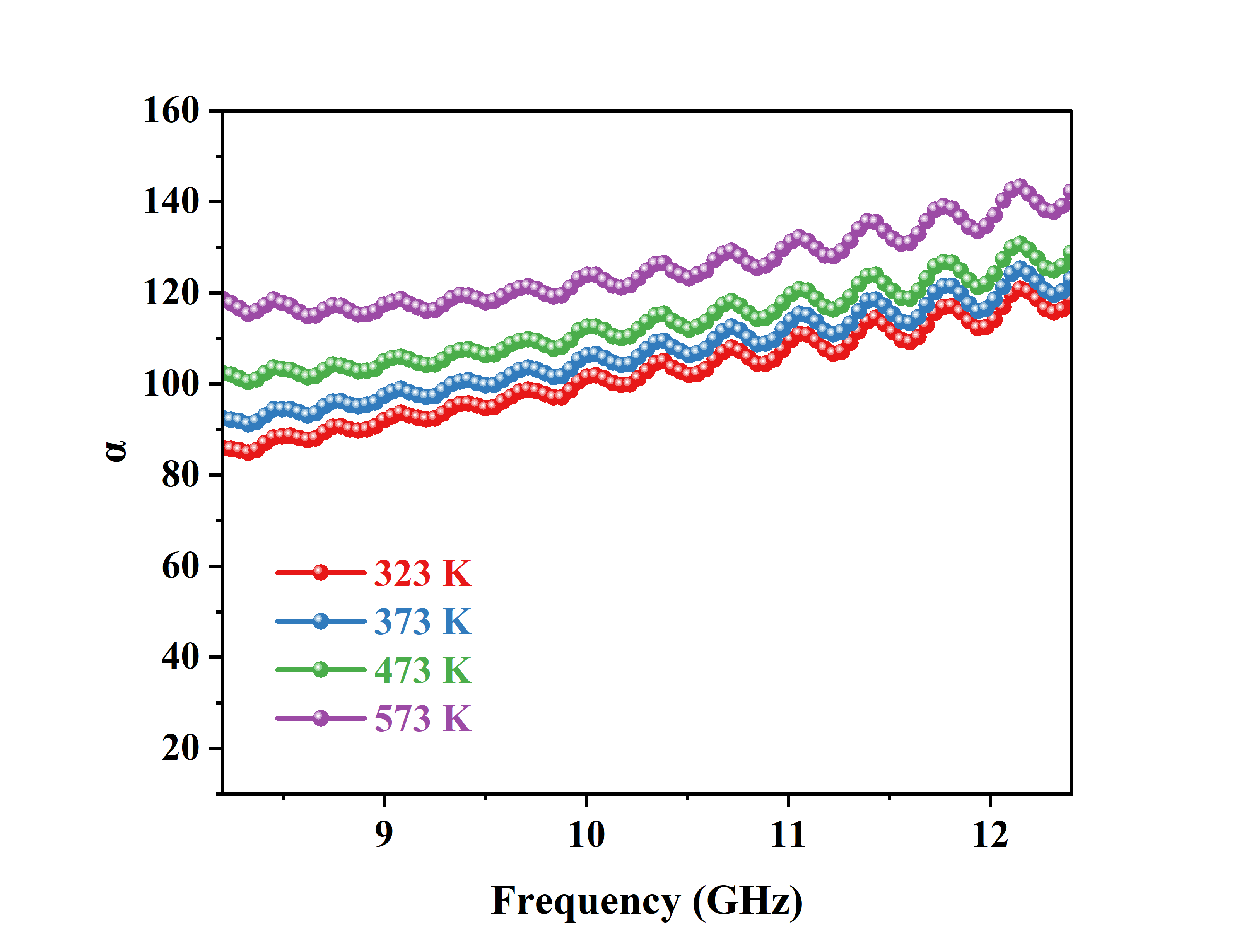


**Figure S13.** Attenuation coefficient (*α*) at different temperatures.


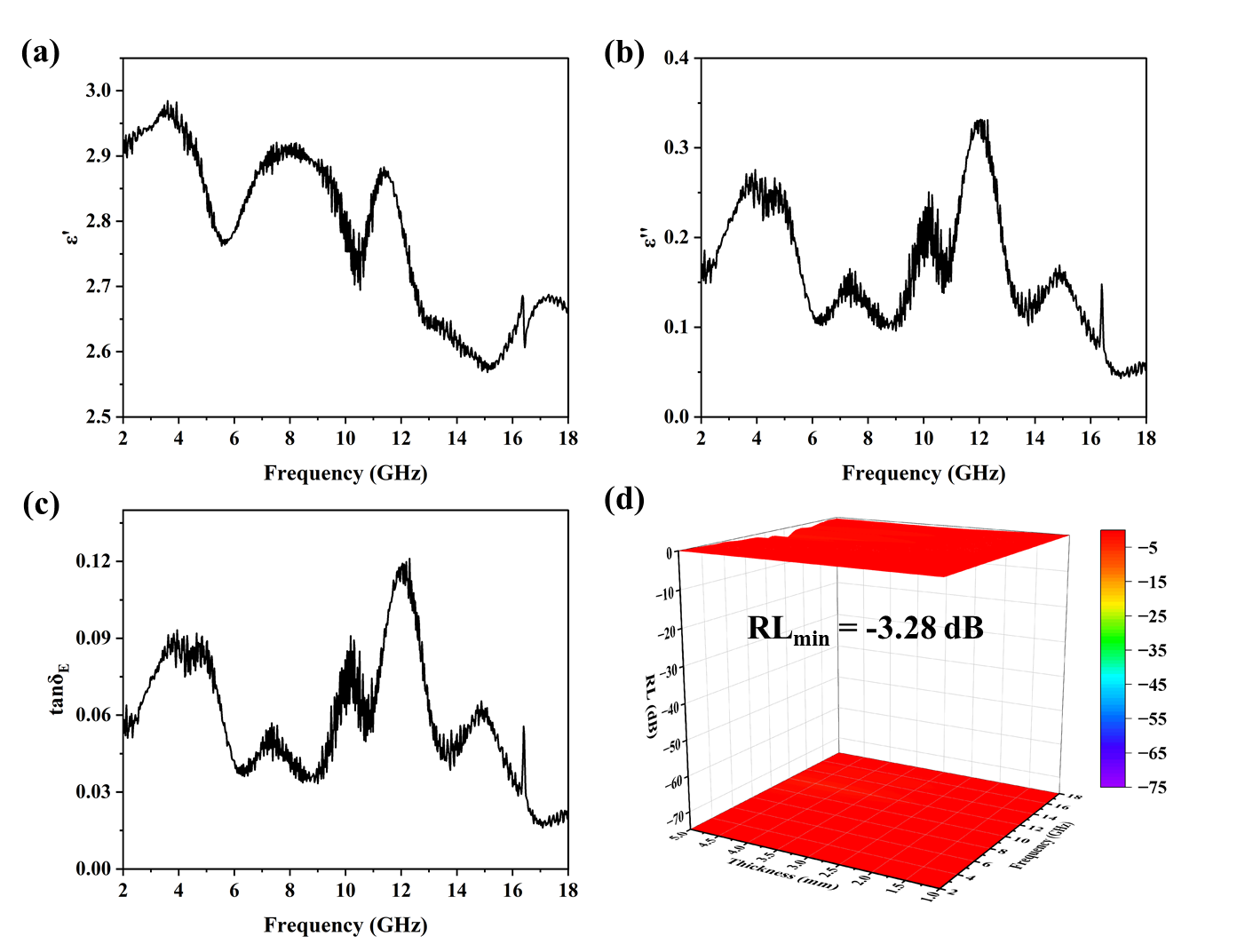


**Figure S14.** a) The real part (ε′) and b) imaginary part (ε′′) of permittivity. c) Dielectric loss tangent (tanδ_E_). d) 3D RL curves (1–5 mm) of PEEK.

**
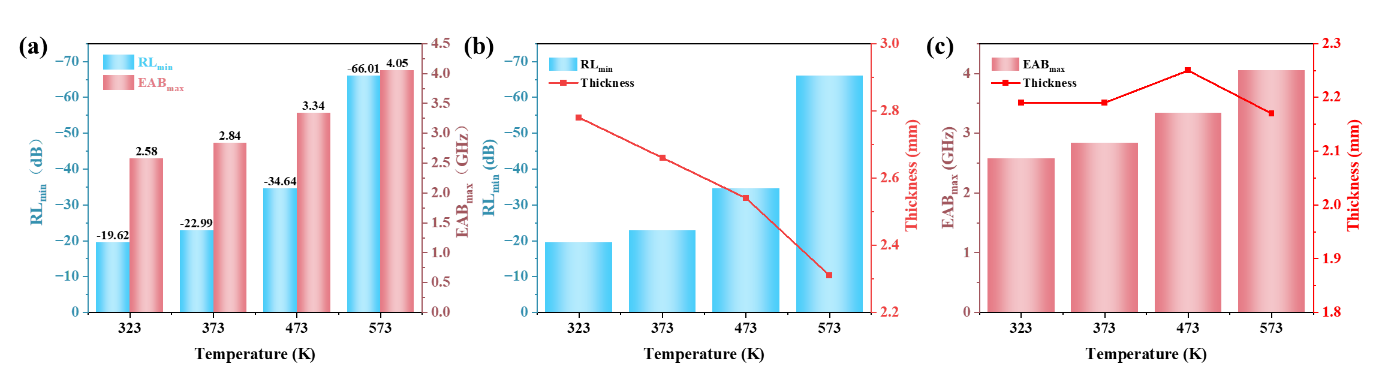
**

**Figure S15.** a) RL_min_ and EAB_max_ at different temperatures. b) RL_min_ and corresponding matching thickness at various temperatures. c) EAB_max_ and corresponding matching thickness under different temperatures of FCGP-25.

**
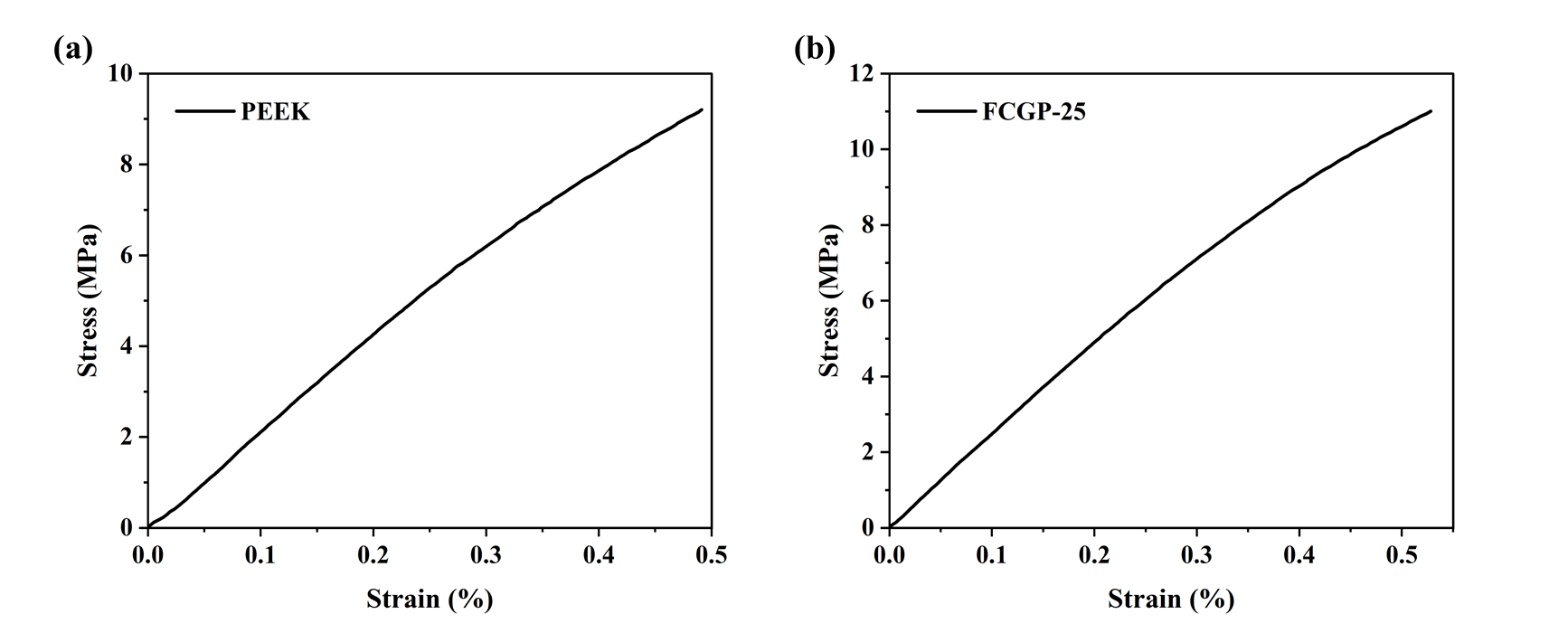
**

**Figure S16.** Stress–strain curves of a) PEEK and b) FCGP-25 composites.

**6. Supplementary Tables**

**Table S1.** EMW absorption performance of different PEEK-based composites at room temperature.

| Samples | RL_min_ (dB) | Thickness(mm) | *f* (GHz) | EAB_max_ (GHz) | Range (GHz) |
| --- | --- | --- | --- | --- | --- |
| FCGP-20 | -16.65 | 5.000 | 5.18 | 2.62 | 15.38~18.00 |
| **FCGP-25** | **-66.62** | **3.330** | **6.46** | **4.58** | **13.36~17.94** |
| FCGP-30 | -74.45 | 2.335 | 8.18 | 4.38 | 13.54~17.96 |
| FCP-25 | -59.21 | 2.695 | 7.28 | 3.84 | 13.92~17.76 |
| FGP-25 | -12.94 | 5.000 | 6.71 | 1.84 | 6.45~8.29 |
| FP-25 | -24.87 | 2.980 | 10.65 | 6.25 | 11.28~17.53 |

**Table S2.** Comparison of EMW absorption performance between FCGP-25 composites and other polymer-based absorbers.

| Sample | Matrix | Loading  (wt %) | RL_min_  (dB) | Thickness  (mm) | EAB  (GHz) | Ref. |
| --- | --- | --- | --- | --- | --- | --- |
| EG/ZnO | Epoxy | 24 | -46.3 | 1.8 | 2.71 | [13] |
| BCN | Natural rubber | 2.8 | -54.24 | 2.5 | 4.16 | [14] |
| MXene/Co/PVDF | PVDF | 12 | -45.6 | 4.0 | 2.2 | [15] |
| MDCF@BN | Epoxy | 25 | -52.77 | 3.0 | 5.6 | [16] |
| SiCnw/BN | Epoxy | 16.69 | -21.5 | 3.0 | 2.8 | [17] |
| Co/MWCNT/PE | PE | 12 | -55 | 4.0 | 5.0 | [18] |
| PMMA@Mxene@Co_3_O_4_ | PMMA | 40 | -52.88 | 2.5 | 5.28 | [19] |
| GNS/EP-CE | EP-CE | 3 | -21.4 | 4.0 | 0.8 | [20] |
| GF/interlayer/CF | Glass/epoxy | 80 | -54.09 | 5.0 | 3.86 | [21] |
| PEEK-50/C-40/FeCo-10 | PEEK | 50 | -35.9 | 1.5 | 3.6 | [22] |
| CNTs/PMMA | PMMA | 8 | -30.2 | / | 3.2 | [23] |
| CFS/EB | Epoxy | 3.5 | -59.2 | 1.95 | 5.82 | [24] |
| **Fe_3_O_4_/CNTs/rGO/PEEK** | **PEEK** | **25** | **-66.62** | **3.33** | **4.58** | **This work** |

**7. Supplementary References**

[1] a) L. Wang, Y. Wang, D. Liu, X. Zhang, G. Wen, X. Zhang, X. Huang, Fabrication of hexagonal platelets of Fe_3_O_4_/Fe_2_O_3_/C modified with polyacrylamide for excellent electromagnetic wave absorption, *Colloids Surf., A* **2025**, 704; b) G. Li, S. Ma, Z. Li, Y. Zhang, Y. Cao, Y. Huang, Temperature‐Induced Self‐Decomposition Doping of Fe_3_GeTe_2_ to Achieve Ultra‐High Tc of 496 K for Multispectral Compatible Strong Electromagnetic Wave Absorption, *Adv. Funct. Mater.* **2023**, 33; c) Q. Song, F. Ye, L. Kong, Q. Shen, L. Han, L. Feng, G. Yu, Y. Pan, H. Li, Graphene and MXene Nanomaterials: Toward High‐Performance Electromagnetic Wave Absorption in Gigahertz Band Range, *Adv. Funct. Mater.* **2020**, 30.

[2] a) Y. Li, X. Liu, X. Nie, W. Yang, Y. Wang, R. Yu, J. Shui, Multifunctional Organic–Inorganic Hybrid Aerogel for Self‐Cleaning, Heat‐Insulating, and Highly Efficient Microwave Absorbing Material, *Adv. Funct. Mater.* **2018**, 29; b) Z. Jiao, J. Hu, M. Ma, Y. Liu, J. Zhao, X. Wang, S. Luan, L. Zhang, One-dimensional core-shell CoC@CoFe/C@PPy composites for high-efficiency microwave absorption, *J Colloid Interface Sci* **2023**, 650, 2014.

[3] Y. Xia, W. Gao, C. Gao, A Review on Graphene‐Based Electromagnetic Functional Materials: Electromagnetic Wave Shielding and Absorption, *Adv. Funct. Mater.* **2022**, 32.

[4] a) S. Wang, X. Zhang, S. Hao, J. Qiao, Z. Wang, L. Wu, J. Liu, F. Wang, Nitrogen-Doped Magnetic-Dielectric-Carbon Aerogel for High-Efficiency Electromagnetic Wave Absorption, *Nanomicro Lett* **2023**, 16, 16; b) Z. Cai, Y. Ma, K. Zhao, M. Yun, X. Wang, Z. Tong, M. Wang, J. Suhr, L. Xiao, S. Jia, X. Chen, Ti_3_C_2_T_x_ MXene/graphene oxide/Co_3_O_4_ nanorods aerogels with tunable and broadband electromagnetic wave absorption, *Chem. Eng. J.* **2023**, 462.

[5] J. Qian, B. Du, M. Cai, C. He, X. Wang, H. Xiong, A. Shui, Preparation of SiC Nanowire/Carbon Fiber Composites with Enhanced Electromagnetic Wave Absorption Performance, *Adv. Eng. Mater.* **2021**, 23.

[6] a) Z. Tang, L. Xu, C. Xie, L. Guo, L. Zhang, S. Guo, J. Peng, Synthesis of CuCo_2_S_4_@Expanded Graphite with crystal/amorphous heterointerface and defects for electromagnetic wave absorption, *Nat. Commun.* **2023**, 14; b) X. Zhang, J. Qiao, Y. Jiang, F. Wang, X. Tian, Z. Wang, L. Wu, W. Liu, J. Liu, Carbon-Based MOF Derivatives: Emerging Efficient Electromagnetic Wave Absorption Agents, *Nanomicro Lett* **2021**, 13, 135.

[7] a) G. Gou, F. Meng, H. Wang, M. Jiang, W. Wei, Z. Zhou, Wheat straw-derived magnetic carbon foams: In-situ preparation and tunable high-performance microwave absorption, *Nano Res.* **2019**, 12, 1423; b) F. Wang, W. Gu, J. Chen, Y. Wu, M. Zhou, S. Tang, X. Cao, P. Zhang, G. Ji, The point defect and electronic structure of K doped LaCo_0.9_Fe_0.1_O_3_ perovskite with enhanced microwave absorbing ability, *Nano Res.* **2021**, 15, 3720; c) H. Zhao, Y. Cheng, Z. Zhang, B. Zhang, C. Pei, F. Fan, G. Ji, Biomass-derived graphene-like porous carbon nanosheets towards ultralight microwave absorption and excellent thermal infrared properties, *Carbon* **2021**, 173, 501.

[8] L. Wu, C. Zhang, W. You, Y. Qian, M. Liu, L. Rao, C. Yang, L. Yang, H. Cao, R. Che, Well‐Aligned Magnetic Cubes for High‐Frequency Magnetic Modulation, *Adv. Funct. Mater.* **2025**, DOI: 10.1002/adfm.202424594.

[9] X. Wang, Y. Lu, T. Zhu, S. Chang, W. Wang, CoFe_2_O_4_/N-doped reduced graphene oxide aerogels for high-performance microwave absorption, *Chem. Eng. J.* **2020**, 388.

[10] a) M. Green, Z. Liu, P. Xiang, X. Tan, F. Huang, L. Liu, X. Chen, Ferric metal-organic framework for microwave absorption, *Mater. Today Chem.* **2018**, 9, 140; b) R. Zhao, M. E. Khalifa, M. M. Hessien, S. M. El-Bahy, T. Li, Y. Ma, Fabrication of carbon fibers doped with Prussian blue derivative composites for enhanced electromagnetic wave absorption, *Adv. Compos. Hybrid Mater.* **2024**, 7; c) Z. Fu, C. Lin, X. Meng, Three dimension Ni_0.5_Zn_0.5_Fe_2_O_4_/BaFe_12_O_19_@carbon composite for light weight, strong absorption and broadband microwave absorbents, *Ceram. Int.* **2021**, 47, 16070.

[11] a) T. Wang, W. Zhao, Y. Miao, A. Cui, C. Gao, C. Wang, L. Yuan, Z. Tian, A. Meng, Z. Li, M. Zhang, Enhancing Defect-Induced Dipole Polarization Strategy of SiC@MoO_3_ Nanocomposite Towards Electromagnetic Wave Absorption, *Nanomicro Lett* **2024**, 16, 273; b) X. Liu, Y. Duan, N. Wu, G. Li, Y. Guo, J. Liu, N. Zhu, Q. Wang, L. Wang, Z. Xu, H. Wei, G. Wang, Z. Zhang, S. Zhang, W. Zhou, T. Ma, T. Wang, Modulating Electromagnetic Genes Through Bi-Phase High-Entropy Engineering Toward Temperature-Stable Ultra-Broadband Megahertz Electromagnetic Wave Absorption, *Nanomicro Lett* **2025**, 17, 164; c) G. Wang, C. Li, D. Estevez, P. Xu, M. Peng, H. Wei, F. Qin, Boosting Interfacial Polarization Through Heterointerface Engineering in MXene/Graphene Intercalated-Based Microspheres for Electromagnetic Wave Absorption, *Nanomicro Lett* **2023**, 15, 152.

[12] a) M.-S. Cao, W.-L. Song, Z.-L. Hou, B. Wen, J. Yuan, The effects of temperature and frequency on the dielectric properties, electromagnetic interference shielding and microwave-absorption of short carbon fiber/silica composites, *Carbon* **2010**, 48, 788; b) J. Xue, Y. Gao, F. Li, Y. Wang, C. Wang, F. Yang, X. Fan, L. Cheng, A novel oxide ceramic matrix composite with integrated high-temperature EMW absorption and mechanical performance, *J. Alloys Compd.* **2024**, 1004.

[13] S. K. Singh, M. J. Akhtar, K. K. Kar, Impact of Al_2_O_3_, TiO_2_, ZnO and BaTiO_3_ on the microwave absorption properties of exfoliated graphite/epoxy composites at X-band frequencies, *Composites, Part B* **2019**, 167, 135.

[14] P. Mou, J. Zhao, G. Wang, S. Shi, G. Wan, M. Zhou, Z. Deng, S. Teng, G. Wang, BCN nanosheets derived from coconut shells with outstanding microwave absorption and thermal conductive properties, *Chem. Eng. J.* **2022**, 437.

[15] R. Li, Q. Gao, H. Xing, Y. Su, H. Zhang, D. Zeng, B. Fan, B. Zhao, Lightweight, multifunctional MXene/polymer composites with enhanced electromagnetic wave absorption and high-performance thermal conductivity, *Carbon* **2021**, 183, 301.

[16] Y. Qian, Y. Tao, Y. Li, J. Hao, C. Xu, W. Yan, Q. Jiang, Y. Luo, J. Yang, High performance epoxy resin with efficient electromagnetic wave absorption and heat dissipation properties for electron packaging by modification of 3D MDCF@hBN, *Chem. Eng. J.* **2022**, 441.

[17] D. Pan, G. Yang, H. M. Abo-Dief, J. Dong, F. Su, C. Liu, Y. Li, B. Bin Xu, V. Murugadoss, N. Naik, S. M. El-Bahy, Z. M. El-Bahy, M. Huang, Z. Guo, Vertically Aligned Silicon Carbide Nanowires/Boron Nitride Cellulose Aerogel Networks Enhanced Thermal Conductivity and Electromagnetic Absorbing of Epoxy Composites, *Nanomicro Lett* **2022**, 14, 118.

[18] M. A. Kazakova, N. V. Semikolenova, E. Y. Korovin, V. A. Zhuravlev, A. G. Selyutin, D. A. Velikanov, S. I. Moseenkov, A. S. Andreev, O. B. Lapina, V. I. Suslyaev, M. A. Matsko, V. A. Zakharov, J.-B. d. E. d. Lacaillerie, Co/multi-walled carbon nanotubes/polyethylene composites for microwave absorption: Tuning the effectiveness of electromagnetic shielding by varying the components ratio, *Compos. Sci. Technol.* **2021**, 207.

[19] J. Guo, Y. Wang, L. Wang, B. Ding, Y. Wang, Y. Sun, S. Dai, D. Wang, S. Bi, High Absorption of Electromagnetic Waves Based on 3D PMMA@Mxene@Co_3_O_4_ Composite Microsphere, *Materials (Basel)* **2024**, 17.

[20] F. Ren, G. Zhu, Y. Wang, X. Cui, Microwave absorbing properties of graphene nanosheets/ epoxy-cyanate ester resins composites, *J. Polym. Res.* **2014**, 21.

[21] H. Zhao, Z. Zhu, Y. Xu, Z. Wang, J. Zhou, Design and preparation of an epoxy resin matrix composite structure with broadband wave-absorbing properties, *Results Phys.* **2024**, 57.

[22] S. Wu, P. Xu, S. Wang, D. Qi, L. Wang, L. Meng, S. Ge, X. Yue, Facile Preparation of Light‐Weight Polyetheretherketone/Graphite/FeCo Ternary Heterogeneous Composite Foam with High Compressive Strength and Excellent Integrated Electromagnetic Wave Absorption Performance, *Macromol. Mater. Eng.* **2022**, 307.

[23] D. Zhou, H. Yuan, Z. Yu, W. Guo, Y. Xiong, G. Luo, Q. Shen, Broadband electromagnetic absorbing performance by constructing alternate gradient structure (AGS) for PMMA-based foams, *Composites, Part A* **2021**, 149.

[24] J. Luo, Z. Lv, Q. Wang, L. Zhang, Y. Zhong, H. Xu, Z. Mao, A wood-inspired epoxy-based electronic packaging material with vertically aligned thermally conductive channels for electromagnetic waves absorption and thermal management, *Chem. Eng. J.* **2024**, 495.
